# Occupational differences in working life expectancy and working years lost in Nordic countries

**DOI:** 10.5271/sjweh.4239

**Published:** 2025-09-01

**Authors:** Karina Undem, Taina Leinonen, Daniel Falkstedt, Gun Johansson, Jacob Pedersen, Eira Viikari-Juntura, Ingrid Sivesind Mehlum, Svetlana Solovieva

**Affiliations:** 1 National Institute of Occupational Health (STAMI), Oslo, Norway.; 2 Institute of Health and Society, University of Oslo, Oslo, Norway; 3 Finnish Institute of Occupational Health, Helsinki, Finland.; 4 Unit of Occupational Medicine, Institute of Environmental Medicine, Karolinska Institute, Stockholm, Sweden.; 5 National Research Centre for the Working Environment, Copenhagen, Denmark; 6 Copenhagen University Hospital – Bispebjerg and Frederiksberg, Copenhagen, Denmark.; 7 Department of Public Health, University of Copenhagen, Copenhagen, Denmark.

**Keywords:** disability retirement, early retirement, employment, extending working life, occupational group, old-age retirement, sickness absence, unemployment, work participation

## Abstract

**Objective:**

Risk of exit from work is both occupation- and country-specific. This study investigated occupational differences in working life expectancy (WLE) and reasons for working years lost (WYL) among employed workers in three Nordic countries.

**Methods:**

We utilized registry-based cohorts of the employed population in Denmark (N=2 383 657), Finland (N=1 266 705) and Norway (N=1 761 166) to estimate WLE for ages 30–65 using the Sullivan method with 2015 data. We further estimated WYL due to sickness absence, unemployment, disability retirement, old-age retirement and other reasons. The analyses were stratified by gender and major occupational group (1^st^ digit in the ISCO-88 code).

**Results:**

Occupational differences in WLE and WYL were observed in all countries. The overall pattern across the countries showed that legislators, senior officials and managers and professionals generally had high WLE, while service and sales workers and employees in manual occupations tended to have lower WLE, with employees in elementary occupations performing the worst. Reasons for WYL varied with country. In general, disability retirement was a significant factor in Denmark, unemployment in Finland, and sickness absence in Norway.

**Conclusion:**

A similar occupational pattern in WLE was observed across the countries, with some occupational groups consistently showing high or low WLE. However, the magnitude of occupational differences in WLE and the reasons for WYL varied across the countries.

Population ageing and the subsequent expected increase in the ratio of pensioners to working individuals are causing concerns for the financial sustainability of welfare states (1). On average, the number of elderly people (≥65 years) per 100 people of working age (20–64) is expected to increase from 31 in 2022 to 54 in 2052 in OECD countries (2). As a result, it has become a political objective in the Nordic countries to reduce temporary exits from work, such as sickness absence, and to extend the length of working life by delaying the time of permanent exit from work (3, 4).

However, the working population is heterogeneous, with various subgroups that differ in their work participation. Previous studies have shown that workers exposed to harmful occupational exposures (eg, heavy physical work or low job control) and workers in low-class occupations (eg, manual and unskilled occupations) have lower work participation (5–7) and a higher risk of exit from work (8–10). This applies particularly to health-related exit paths (9) but may also hold for unemployment and non-health-related exits (11, 12). Furthermore, different pathways of exit from work have somewhat different occupational distributions. For instance, the feasibility of early retirement may be constrained for individuals in low-class occupations due to limited pension savings or access to pension schemes (9). It is, therefore, beneficial to consider multiple exit routes. Furthermore, considering a broad time span of an individual’s working life, and taking into account both temporary and permanent exits from work, offers a more comprehensive understanding of work participation throughout the working age.

Measures of working life expectancy (WLE) can take different exit routes into account and provide an estimate of the expected length of working life (13). Depending on the definition of working life, WLE can, for instance, be applied to quantify the years a person at a specific age is expected to be economically active (ie, employed or unemployed) (14) or years expected to be employed (15). Conversely, working years lost (WYL) can be defined as the number of years a person is expected not to be economically active or employed.

WLE and WYL have been studied in relation to physical workload and occupational class (6, 7, 15–18). However, previous studies have typically included a limited number of occupational groups, often based on social class, and only a few have examined more specific groups (19). Including a more extensive set of occupational groups, where the workers within the groups are more similar in terms of education and working conditions, will help identify occupations with a high potential for workplace interventions.

Moreover, there is a lack of cross-country comparative studies. Labor market policies and social insurance systems can influence work participation and age of exit from work, as well as the predominant work exit routes (20). Comparisons across countries may enhance the understanding of the interplay between country-specific features, occupation-specific factors and work participation.

The aim of the present study was to estimate WLE and WYL among employed workers in Nordic countries and examine occupational similarities and differences within and across the countries. WLE is here defined as the number of years a person at a specific age is expected to be in work, not receiving any social benefits indicating that he or she is temporarily absent from work (eg, sickness benefits). WYL is defined as expected time outside of work due to sickness absence, unemployment, disability retirement, old-age retirement and other reasons. We examined WLE and WYL in Denmark, Finland and Norway, as available data were considered comparable for these three countries.

## Methods

### Data sources

The study utilized data from 2015, drawing upon three national registry-based cohorts. The data comprised information on gender, birth year, occupation and mortality, as well as episodes of employment and recipiency of social benefits related to absence from work.

The *Danish cohort of employees in 2015* includes all employees in Denmark as of November 2014. Data are drawn from the Danish Registry-based Labor Force Statistics (RAS) and linked with social benefits data from the Danish Register for the Evaluation of Marginalization (DREAM). Mortality information was obtained from the Danish Death Register.

The *Finnish Nationwide Working-Age Cohort 2015* consists of a randomly selected 70% sample of the residents of Finland. Data are sourced from the Finnish Social Insurance Institution, the Finnish Centre for Pensions and the Finnish Longitudinal Employer-Employee Data of Statistics Finland. Mortality information was obtained from the Population Census register and supplied by the Finnish Social Insurance Institution.

The *Norwegian Working Age Cohort* includes individuals born 1930–1992 and residing in Norway 2000-2010. All data applied in this study are sourced from different statistical data sources and administrative registers obtained through Statistics Norway, including the event database FD-Trygd, which contains longitudinal information on all employment periods and social benefits.

### Study population

The study included individuals aged 30–64 years who were employed in November (Denmark and Norway) or December (Finland) 2014 and resided in the respective countries in 2015 (Denmark N=2 383 657; Finland N=1 266 705; Norway N=1 750 132). Age was measured at the beginning of the year in Denmark and at the end of the year in Finland and Norway. Self-employed individuals were excluded in Finland and Norway. For occupation-specific analyses, we excluded armed forces occupations and individuals missing information on occupation or who had an occupational code that could not be converted to the ISCO-88 (COM) (Denmark N=715 194; Finland N=7552; Norway N=70 809).

### Labor market states

**Figure 1:**
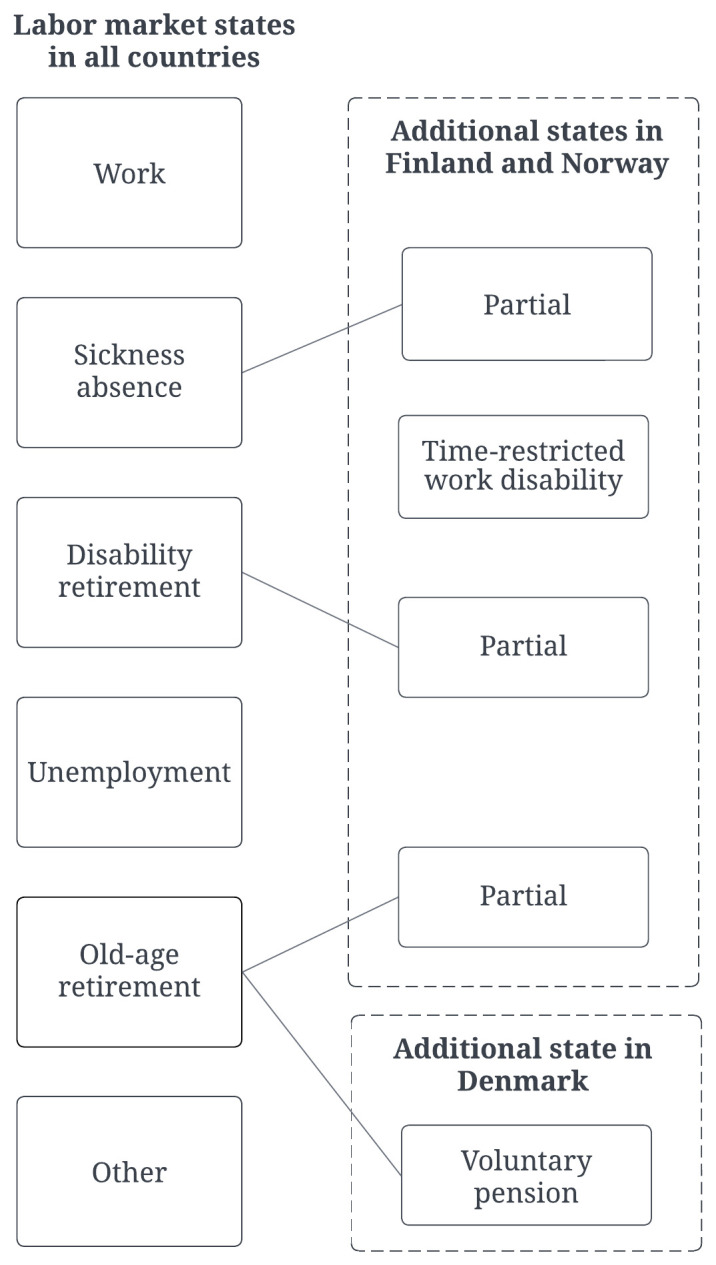
Labor market states in Denmark, Finland and Norway.

Due to differences in labor market policies and social insurance systems, as well as differences in data availability, the number of labor market states differed across the countries. Six overall labor market states were defined: *work*, *sickness absence*, *unemployment*, *disability retirement*, *old-age retirement*, and *other*. For Finland and Norway, four additional states were included: *partial sickness absence*, *partial disability retirement*, *partial old-age retirement* and *time-restricted work disability*. For Denmark, one extra state was included: *voluntary pension*. This resulted in seven labor market states for Denmark and ten for Finland and Norway (figure 1).

Time spent in the various labor market states during 2015 was calculated for each person. The Danish data consisted of weekly registrations, and overlaps between the labor market states were solved and recorded in DREAM according to the pre-existing hierarchy in the register (21). In Finland and Norway, event data were utilized, consisting mainly of daily records (start and stop dates), with the exception of pensions which are typically paid monthly in both countries. To solve overlaps between labor market states in Finland and Norway, a hierarchy was developed based on the accuracy of data sources and the pre-existing hierarchy in the Danish data: (i) old-age retirement, (ii) partial old-age retirement, (iii) disability retirement, (iv) partial disability retirement, (v) time-restricted work disability, (vi) sickness absence, (vii) partial sickness absence, (viii) unemployment, (ix) work, (x) other.

*Work* was defined as either full-time or part-time work. For Finland and Norway, individuals were in work when they had an employment period and did not receive any social benefits or pensions that fall under the definition of the other labor market states. For Denmark, information on employment periods was lacking, and individuals were assumed to be in work when they did not receive any social benefits or pensions.

*Sickness absence* was defined as receiving sickness benefits provided by the social insurance agencies of the respective countries. In all countries, the salary or compensation is usually paid by the employer during the first weeks of the sickness absence: 30 calendar days in Denmark, 10 weekdays (including Saturdays) in Finland, and 16 calendar days in Norway. For Finland and Norway, these periods were not included in the sickness absence state. For Denmark, these days were included, however, only if the sickness absence episode exceeded the employer-covered period.

In Denmark, sickness absence benefits are available to employees who have worked a minimum of 74 hours for an employer within eight weeks. While there was no time limit on the duration of sickness benefits in 2015, payment was commonly stopped after 22 weeks. Permanent residents in Finland are eligible for sickness absence benefits for up to 300 weekdays (Sundays excluded). In Norway, individuals who have been employed for a minimum of four weeks preceding their sickness absence are eligible for benefits, which can be received for a maximum of 52 weeks.

In Finland and Norway, sickness absences with grades equal to 50% (possible in both countries) or lower (only Norway) were included in the *partial sickness absence* state. For Denmark, graded sickness absences were included in the overall sickness absence state as information on grade was unavailable.

*Time-restricted work disability* was defined as receiving vocational rehabilitation benefits or full or partial temporary disability pension (Finland) and work assessment allowance (Norway).

*Disability retirement* was defined as receiving a disability pension. Individuals in Denmark with a permanent work capacity reduction can receive disability pension, usually provided until age 65. For Denmark, this state also includes individuals with partly reduced work capacity, eg, individuals eligible for a Flexjob (22). In Finland, individuals whose work ability has been reduced for at least one year due to an illness, injury or handicap, may receive a disability pension. Only permanent pensions were included in this state. Individuals in Norway with a permanent reduction in their work capacity due to disease or injury may be entitled to disability pension. In Finland and Norway, disability pensions with degrees of 50% (possible in both countries) or below (only Norway) were included in the *partial disability retirement* state.

*Unemployment* was defined as receiving unemployment benefits. Individuals in Denmark without or with a highly reduced wage income may apply for insurance-based or state-based unemployment benefits. The state-based benefit is without a time limit. In Finland, unemployed or laid-off individuals registered as job seekers may receive unemployment benefits. The minimum-level benefit can be received without time limit. Unemployed or temporarily laid-off individuals residing in Norway with previous employment may receive unemployment benefits for up to two years.

The *old-age retirement* state included recipiency of any type of old-age pension or early-old-age pension. In 2015, old-age pension could be received from age 65 in Denmark. Individuals in Finland could receive an old-age pension flexibly at 63–68 years. Additionally, there were special early pensions for farmers. In Norway individuals had the option of retiring from the age of 62 (on contractual early retirement pension and/or state retirement pension), given sufficient pension savings. In Finland and Norway, pension degrees ≤50% were included in the *partial old-age retirement* state. In Finland, the partial pension could be received from ≥61 years.

In Denmark, individuals receiving payments from the insurance-based scheme “efterløn” were included in the *voluntary early retirement* state. Members of the early retirement scheme can retire up to five years before the official retirement age (65 years in 2015), provided that they meet certain requirements, such as having made monthly payments into the scheme for at least thirty years (23). In addition, the scheme offers a bonus to those who choose to postpone voluntary early retirement for one to five years. The early retirement scheme is co-financed by the state and has no health-related eligibility criteria.

The state *other* included individuals outside of work who were not placed in any of the abovementioned labor market states, such as students and individuals on parental leave.

### Occupational groups

Occupations in Denmark were coded according to Statistics Denmark’s Classification of Occupations (DISCO-08). To ensure smoother Nordic comparisons, the occupational codes were converted to DISCO-88 utilizing a crosswalk facilitated by Statistics Denmark (24). However, all occupations could not be converted, as one-to-one translation was missing for approximately 18% of the occupations in the crosswalk. In Finland and Norway, occupations were classified according to the Classification of Occupations 2001 by Statistics Finland (FISCO-01) (25) and the Norwegian Standard Classification of Occupations (STYRK-98) (26), respectively, both based on ISCO-88 (COM). To enhance the comparability of occupational groups across the countries, the respective national codes were converted to ISCO-88 (COM) using a Nordic crosswalk (27).

Nine major occupational groups were created based on the first digit of ISCO-88 (COM): (i) legislators, senior officials and managers; (ii) professionals; (iii) technicians and associate professionals; (iv) clerks; (v) service workers and shop and market sales workers; (vi) skilled agricultural and fishery workers; (vii) craft and related trades workers; (viii) plant and machine operators and assemblers; and (ix) elementary occupations.

### Estimation of WLE and WYL

The Sullivan method (28) was used to estimate WLE and WYL for ages 30–65. That is, we combined age-specific prevalence rates of labor market states and period life tables based on data from one year (2015) as follows: For each one-year age group of 30–64 years, we calculated the mean proportion of time in 2015 spent in different labor market states stratified by country, gender, and occupational group. One-year age-specific mortality rates were calculated for the total and the occupation-specific male and female study populations and used to construct period life tables estimating the number of person-years lived for each age. The expected number of years spent in each labor market state until age 65 was subsequently estimated by combining the age-specific labor market rates with the corresponding person-years lived from the period life tables. The estimations were done separately for men and women, and by country and occupational group.

WLE represents the number of years expected to be in work until age 65. Thus, the theoretical maximum WLE after age 30 is 35 years. WYL denotes the expected number of years outside of work due to sickness absence, unemployment, disability retirement, old-age retirement and other reasons until age 65. Specifically, WYL calculated at age 30 takes into account expected time outside of work at 30–65 years, thereby also including time spent in old-age retirement that occurs at the upper end of this age range. WLE and WYL were truncated at age 65 as this was the official retirement age in Denmark in 2015.

## Results

The occupational distributions in Denmark, Finland and Norway, had some minor variations (table 1). Denmark had somewhat more employees working as professionals, whereas Finland had relatively many skilled agricultural and fishery workers and craft and related trades workers. Legislators were most common in Norway.

**Table 1:** Size of occupational group, by gender and country.

	Denmark			Finland			Norway	
	Men N (%)	Women N (%)		Men N (%)	Women N (%)		Men N (%)	Women N (%)
Total (individuals with known occupation)	911 712 (100)	949 770 (100)		622 899 (100)	636 254 (100)		856 652 (100)	822 671 (100)
Legislators, senior officials and managers	61 489 (6.7)	26 115 (2.7)		48 540 (7.8)	19 728 (3.1)		114 978 (13.4)	64 511 (7.8)
Professionals	215 990 (23.7)	334 507 (35.2)		122 166 (19.6)	141 964 (22.3)		169 247 (19.8)	200 249 (24.3)
Technicians and associate professionals	128 531 (14.1)	131 689 (13.9)		114 739 (18.4)	157 182 (24.7)		164 960 (19.3)	195 802 (23.8)
Clerks	52 019 (5.7)	109 607 (11.5)		20 394 (3.3)	63 798 (10)		44 346 (5.2)	68 371 (8.3)
Service workers and shop and market sales workers	115 855 (12.7)	256 286 (27.0)		44 667 (7.2)	161 150 (25.3)		72 152 (8.4)	225 551 (27.4)
Skilled agricultural and fishery workers	9998 (1.1)	2776 (0.3)		29 519 (4.7)	134 30 (2.1)		6925 (0.8)	1972 (0.2)
Craft and related trades workers	151 245 (16.6)	9417 (1.0)		124 614 (20)	12 305 (1.9)		125 743 (14.7)	5790 (0.7)
Plant and machine operators and assemblers	83 956 (9.2)	18 793 (2.0)		88 767 (14.3)	21 323 (3.4)		100 943 (11.8)	15 837 (1.9)
Elementary occupations	92 629 (10.2)	60 580 (6.4)		29 493 (4.7)	453 74 (7.1)		57 358 (6.7)	44 588 (5.4)

At 30–65 years (WLE at age 30), WLE was 26.3 years for Danish men, 30.2 for Finnish men and 28.8 for Norwegian men (supplementary material, www.sjweh.fi/article/4239, table S1). WLE was somewhat lower among women, with 23.7 years in Denmark, 30.0 in Finland and 27.3 in Norway. Similar patterns were seen for WLE at older ages.

Reasons for WYL varied across the countries. In Denmark, when calculating WYL at age 30, most working years were lost due to disability retirement and unemployment (relative measures in figure 2; absolute measures in supplementary table S1). In Finland, the largest contributor was unemployment, and in Norway, relatively many working years were lost due to sickness absence and time-restricted work disability, particularly among women. The leading reasons for WYL changed with the calculation age. For WYL at age 55, the primary reason was old-age retirement for all strata, except for women in Norway, for whom most working years were lost due to disability retirement.

**Figure:**
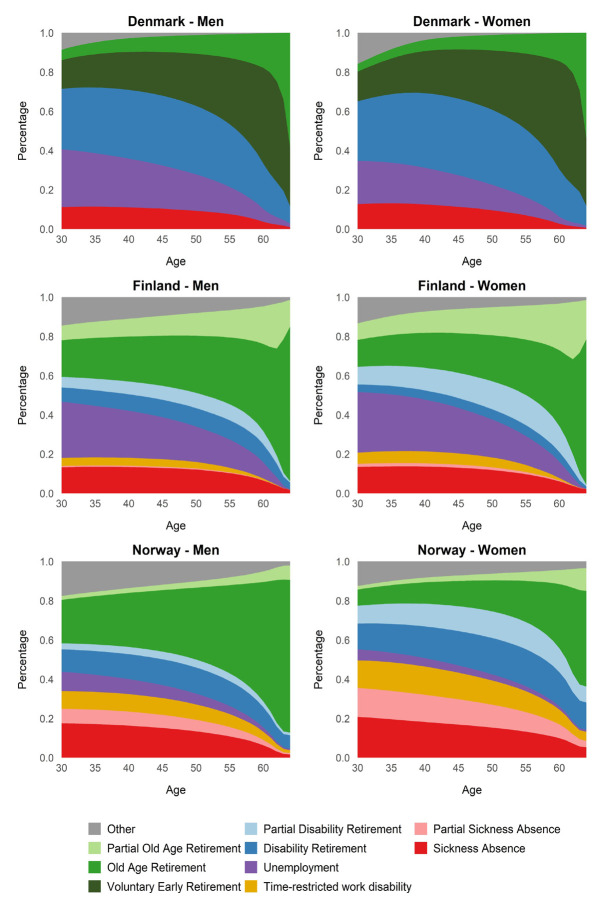
**Figure 2.** Reasons for working years lost (WYL) as a percentage of expected time outside of work until age 65, among the employed population by country and gender. WYL is calculated for each age at 30–64 years and reflects the reasons for expected years outside of work from that age up to 65.

**Figure 3:**
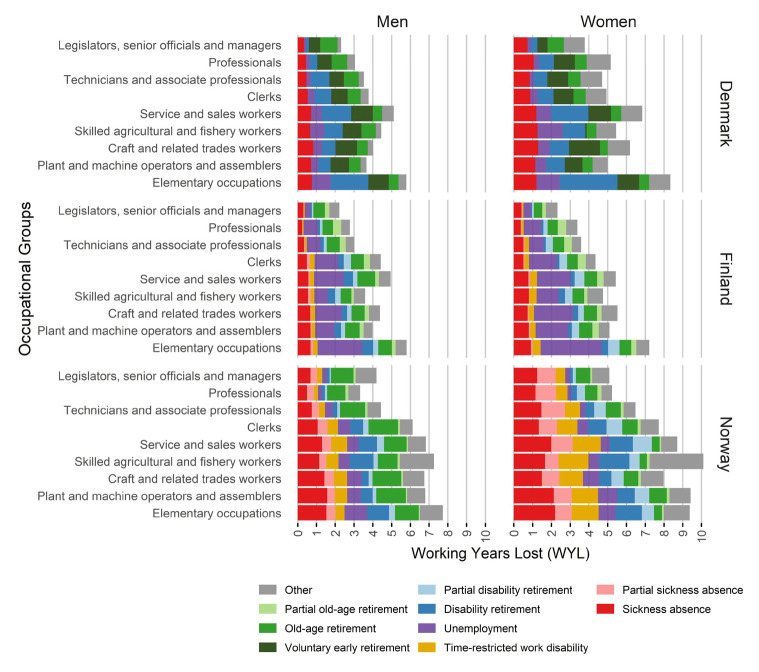
Occupational differences in working years lost (WYL) until age 65 among employed men and women in Denmark, Finland and Norway. WYL is calculated at age 30.

Occupational differences in WLE and WYL were observed in all countries and for both genders. Among men in Norway, WLE at age 30 was highest for professionals (figure 3; supplementary tables S2 and S3). For men in Denmark and Finland, and for women in all countries, legislators, senior officials, and managers represented the occupational group with the highest WLE and fewest WYL. Employees in elementary occupations had the lowest WLE, except for women in Norway, for whom skilled agricultural and fishery workers were expected to spend the least time in work. A similar pattern of occupational differences in WLE was observed at older ages as well (supplementary tables S2 and S3).

The difference in WLE between the occupational group with the lowest and the highest WLE (occupational difference in WLE) was most pronounced in Finland, with an absolute difference of 5.1 years for men and 5.6 years for women at age 30. In comparison, Denmark had a lower occupational difference of 3.5 years for men and 4.7 years for women, while Norway had 4.8 years for men and 5.0 years for women.

In general, occupation-specific reasons for WYL followed those in the general employed population. Yet, some occupational differences were observed (figure 3; supplementary tables S2 and S3). Across all countries, service and sales workers and elementary occupations had relatively many WYL due to disability retirement. Occupational groups requiring higher education had relatively few WYL due to unemployment. When examining the reasons for WYL as a percentage of total WYL, occupational groups requiring higher education had a significantly higher share attributable to old-age retirement compared to other occupational groups (supplementary table S4).

## Discussion

### Summary of main results

WLE for the general employed population was higher among men than women. The leading reasons for WYL varied based on country and age for calculating WYL. In general, disability retirement was a significant cause in Denmark, unemployment in Finland and sickness absence in Norway. Occupational differences in WLE were observed in all countries, with the greatest occupational difference being observed in Finland. Overall, legislators, senior officials and managers and professionals had relatively high WLE, while clerks, service workers and employees in manual occupational groups (major occupational group 6–9) had lower WLE, with employees in elementary occupations performing the worst.

### Strengths and limitations

WLE results are highly sensitive to methodological variations, such as the included study population (eg, age range and employed versus general population), the definition of WLE (eg, being economically active versus employed) and the approach used to estimate WLE (5, 29). A strength of this study was the cross-country harmonization of these aspects, which enabled reasonable comparisons of occupational differences in WLE and WYL across the countries. The Danish study population was effectively one year older than the Finnish and Norwegian (as age was calculated at the beginning of the year in Denmark and the end of the year in Finland and Norway). However, estimations of WLE for ages 31–65 using harmonized age did not significantly change the cross-country WLE and WYL results.

It is important to note that since we used period life tables (Sullivan’s method) to estimate WLE, we assume constant age-specific labor market participation and mortality rates. This warrants caution when interpreting the results, particularly since the study is based on data from a single year (2015). The estimates are not predictions of what is likely to happen. Instead, they indicate what would happen if the conditions of the time of measurement continued in the future. The approach is generally well-suited for comparative analyses, and the results from this study, therefore, provide valuable insights into differences in WLE across occupational groups (7).

A related limitation is that the study relies on relatively old data (from 2015), as more recent data were unavailable. Nordic countries have since seen some variations in the economy and the labor market (30) as well as pension reforms (4), which may have influenced WLE and WYL, particularly in cross-country comparisons. However, even though the absolute estimates of occupation-specific WLE might change, we expect the overall pattern of occupational differences to be persistent over time (31).

Another limitation was the variation in the availability of data across the countries, which caused different under- and over-estimation of time spent in the labor market states. Since only sickness absence episodes expanding beyond the employer-covered period are included in the registries, time spent in sickness absence was somewhat underestimated. More sickness absence episodes were presumably unaccounted for in Denmark than in Finland and Norway, as the employer-covered period is longest in Denmark. However, this was partly levelled out by the fact that, in the Danish data, the calculation of the length of the sickness absences, included the initial employer-covered days. Additionally, for Denmark, WLE may have been overestimated to some extent, as data on employment periods were missing and individuals were assumed to work if they did not receive any type of social benefits or pensions.

Finally, employees with missing occupational information were excluded from the occupation-specific study population. This was mainly an issue in the Danish study population, as some individuals lacked a valid occupational code and as some occupational codes could not be converted from DISCO-08 to DISCO-88. Consequently, the findings from Denmark may be subject to bias. However, the distribution of major occupational codes (table 1) is fairly similar to occupational distributions reported in other sources (eg, 32.).

### Interpretation of the results

Comparisons with previous studies are challenging, as there is a lack of comparability in terms of the study populations (eg, general population versus employed) and methodological aspects (eg, WLE defined as economically active versus in work). Nevertheless, in line with previous research, the present study found cross-country differences in WLE (14, 33). However, as expected time in sickness absence in the present study was included in WYL and not in WLE, the results may be more in line with those from studies looking at healthy working life expectancy (HWLE), ie the number of years expected to be working while in good health (29). Also for HWLE, cross-country differences have been observed (34).

The findings from the present study highlight that even across relatively similar welfare countries such as the Nordics, differences in the economy, labor market policies, and social insurance systems may result in cross-country differences in WLE and reasons for WYL. For instance, while there are early retirement options in Finland and Norway (eg, flexible retirement age and contractual early retirement pension), the *voluntary early retirement* state was included only for Denmark, as the Danish voluntary early retirement scheme has no comparable equivalent in the Finnish or Norwegian systems. The early retirement scheme is an important pathway of exit from work in Denmark (35) and contributes to Denmark having more WYL due to old-age retirement than Finland and Norway (supplementary table S1). Particularly for women, the voluntary pension scheme appears to be an important explanatory factor for cross-country differences. Institutional differences across the countries are also reflected in the fact that the predominant reasons for WYL vary. The relatively high number of WYL due to unemployment in Finland and sickness absence (including time-restricted work disability) in Norway, is fairly aligned with previous studies (6, 36). For Denmark, we found that disability retirement was a relatively more common reason for WYL than previously reported (18). This could be due to how the disability retirement state was defined, which in the present study was defined as a wider concept than previous studies and included, for example, flexjob in Denmark.

A similar pattern of occupational differences in WLE and WYL was present in all countries: clerks, service workers and manual occupational groups tended to have lower WLE compared to occupational groups requiring higher education. The findings align with previous studies reporting lower WLE among manual workers in Finland (6, 7), as well as in some non-Nordic European countries (37–39). The present study contributes to the existing literature by showing that the magnitude of occupational difference in WLE appears to differ across countries, also when similar methodology is employed. Furthermore, the present study shows that some occupational groups have consistently high or low WLE across countries, while other groups have cross-country inconsistencies. This highlights the need for future studies to go beyond broad occupational classes when examining working life participation.

A consistently low WLE across countries, as seen among clerks, service workers and elementary occupations, may indicate that there are common occupation-specific factors driving the results. These occupations often have a high prevalence of potentially harmful occupational exposures, such as low job control, high emotional demands and heavy physical work, that serve as risk factors for several diseases and health problems and may influence the threshold for staying in work while ill (40, 41). However, there is a complex interplay between occupation, health and work participation. The observed occupational difference may also stem from health selection into occupations (42) or variations in other factors across occupational groups, such as lifestyle (43).

The occupational groups with low WLE also tended to have more WYL due to unemployment. For some workers, unemployment may be the path out of work due to poor health or an imbalance between their capacity and the demands of their work. But the results also indicate that a country’s economy and unemployment rate may affect occupational groups differently. Studies from Spain (37) and Italy (38) show that there is an occupational difference in the effects of a recession or financial crisis, and that the decline in WLE is most pronounced in manual occupations.

Finally, there were exceptions to the abovementioned pattern in occupational differences in WLE. For example, Danish plant and machine operators and assemblers and Finnish skilled agricultural and fishery workers had relatively high WLE. These exceptions highlight that country-specific factors are important in understanding occupational differences in WLE and WYL. The occupational distribution, workers’ characteristics (health, lifestyle, motivation), labor market features and social insurance systems may influence occupational differences in WLE and explain differences across countries.

### Implications and future research

The present study shows that some occupational groups have consistently low WLE in all countries, despite cross-country variations in labor markets and social insurance systems. When considering preventive workplace measures to extend working life, these groups may be of particular interest. However, more research is needed to understand whether the occupational differences are due to harmful occupational factors causing lower WLE, or something else, such as selection into occupation or lifestyle.

Furthermore, the findings may indicate that some occupational groups face greater challenges for remaining in work, and that these groups find a pathway out of work depending on country-specific features, ie unemployment in Finland and sickness absence in Norway. More knowledge on the workability of the persons in these occupational groups is vital to determine for whom it will be possible to extend the working life. We encourage future studies to consider multiple reasons for work exit, including unemployment and other types of non-employment.

### Concluding remarks

Occupational differences in WLE were observed in all countries, with the greatest occupational difference being observed in Finland. Overall, legislators, senior officials and managers and professionals had highest WLE, while clerks, service workers and employees in manual occupational groups tended to have lower WLE, with employees in elementary occupations performing the worst. Leading reasons for WYL varied based on country and age for calculating WYL. In general, disability retirement was a significant cause in Denmark, unemployment in Finland and sickness absence in Norway. When considering preventive workplace measures to extend working life, it may be of particular interest to target occupational groups with consistently low WLE across countries.

## Supplementary material

Supplementary material

## References

[1] OECD. Recommendation of the Council on Ageing and Employment Policies. OECD/LEGAL/0419. 2015.

[2] OECD. Pensions at a Glance 2023: OECD and G20 Indicators. Paris: OECD Publishing; 2023. Available from: 10.1787/678055dd-en

[3] Fredriksen D, Holmøy E, Strøm B, Stølen NM. Fiscal effects of the Norwegian pension reform–A micro–macro assessment. J Pension Econ Finance 2019;18(1):88–123. 10.1017/S1474747217000361

[4] Kuivalainen S, Kuitto K. Finland: pension reforms in Finland. The evolution of supplementary pensions: Edward Elgar Publishing; 2022. p. 77–98.

[5] Solovieva S, de Wind A, Undem K, Dudel C, Mehlum IS, van den Heuvel SG et al. Socioeconomic differences in working life expectancy: a scoping review. BMC Public Health 2024 Mar;24(1):735. 10.1186/s12889-024-18229-y 38454363 PMC10921693

[6] Schram JL, Solovieva S, Leinonen T, Viikari-Juntura E, Burdorf A, Robroek SJ. The influence of occupational class and physical workload on working life expectancy among older employees. Scand J Work Environ Health 2021 Jan;47(1):5–14. 10.5271/sjweh.3919 32869106 PMC7801139

[7] Leinonen T, Martikainen P, Myrskylä M. Working Life and Retirement Expectancies at Age 50 by Social Class: Period and Cohort Trends and Projections for Finland. J Gerontol B Psychol Sci Soc Sci 2018 Jan;73(2):302–13. 10.1093/geronb/gbv104 26560805

[8] White M, Wagner S, Schultz IZ, Murray E, Bradley SM, Hsu V et al. Modifiable workplace risk factors contributing to workplace absence across health conditions: A stakeholder-centered best-evidence synthesis of systematic reviews. Work 2013;45(4):475–92. 10.3233/WOR-131628 23531590

[9] Carr E, Fleischmann M, Goldberg M, Kuh D, Murray ET, Stafford M et al. Occupational and educational inequalities in exit from employment at older ages: evidence from seven prospective cohorts. Occup Environ Med 2018 May;75(5):369–77. 10.1136/oemed-2017-104619 29530976 PMC5909745

[10] Knardahl S, Johannessen HA, Sterud T, Härmä M, Rugulies R, Seitsamo J et al. The contribution from psychological, social, and organizational work factors to risk of disability retirement: a systematic review with meta-analyses. BMC Public Health 2017 Feb;17(1):176. 10.1186/s12889-017-4059-4 28178966 PMC5299735

[11] Robroek SJ, Schuring M, Croezen S, Stattin M, Burdorf A. Poor health, unhealthy behaviors, and unfavorable work characteristics influence pathways of exit from paid employment among older workers in Europe: a four year follow-up study. Scand J Work Environ Health 2013 Mar;39(2):125–33. 10.5271/sjweh.3319 22949091

[12] Sundstrup E, Hansen ÅM, Mortensen EL, Poulsen OM, Clausen T, Rugulies R et al. Retrospectively assessed physical work environment during working life and risk of sickness absence and labour market exit among older workers. Occup Environ Med 2018 Feb;75(2):114–23. 10.1136/oemed-2016-104279 28819019 PMC5800344

[13] Shiri R, Hiilamo A, Lallukka T. Indicators and determinants of the years of working life lost: a narrative review. Scand J Public Health 2021 Aug;49(6):666–74. 10.1177/1403494821993669 33645306 PMC8512267

[14] Loichinger E, Weber D. Trends in working life expectancy in Europe. J Aging Health 2016 Oct;28(7):1194–213. 10.1177/0898264316656509 27590798

[15] Schram JL, Schuring M, Oude Hengel KM, Burdorf A, Robroek SJ. The influence of chronic diseases and poor working conditions in working life expectancy across educational levels among older employees in the Netherlands. Scand J Work Environ Health 2022 Jul;48(5):391–8. 10.5271/sjweh.4028 35471244 PMC9527781

[16] Kaprio J, Sarna S, Fogelholm M, Koskenvuo M. Total and occupationally active life expectancies in relation to social class and marital status in men classified as healthy at 20 in Finland. J Epidemiol Community Health 1996 Dec;50(6):653–60. 10.1136/jech.50.6.653 9039385 PMC1060383

[17] Kadefors R, Nilsson K, Östergren PO, Rylander L, Albin M. Social inequality in working life expectancy in Sweden. Z Gerontol Geriatr 2019 Feb;52 Suppl 1:52–61. 10.1007/s00391-018-01474-3 30413944 PMC6373384

[18] Pedersen J, Schultz BB, Madsen IE, Solovieva S, Andersen LL. High physical work demands and working life expectancy in Denmark. Occup Environ Med 2020 Aug;77(8):576–82. 10.1136/oemed-2019-106359 32398291 PMC7402449

[19] Hayward MD, Grady WR. Work and retirement among a cohort of older men in the United States, 1966-1983. Demography 1990 Aug;27(3):337–56. 10.2307/2061372 2397817

[20] Kadefors R, Nilsson K, Rylander L. ÖStergren P-O, Albin M. Occupation, gender and work-life exits: a Swedish population study. Ageing Soc 2018;38(7):1332–49. 10.1017/S0144686X17000083

[21] The Danish Agency for Labour Market and Recruitment (STAR). DREAM vejledning - version 46 [DREAM instructions - version 46] 2021 [cited 21.01.2025. Available from: https://www.dst.dk/Site/Dst/SingleFiles/GetArchiveFile.aspx?fi=6705686690&fo=0&ext=forskning

[22] Udbetaling Danmark. Flexi job scheme 2021 [cited 27.01.2025]. Available from: https://lifeindenmark.borger.dk/pension/flexi-job-scheme

[23] The Danish Agency for Labour Market and Recruitment (STAR). Efterløn [cited 16.12.2024]. Available from: https://star.dk/ydelser/pension-og-efterloen/efterloen

[24] Statistics Denmark. DISCO-88 [cited 16.12.2024]. Available from: https://www.dst.dk/da/Statistik/nyheder-analyser-publ/Publikationer/VisPub?cid=4831

[25] Statistics Finland. Classification of Occupations 2001 [cited 16.12.2024]. Available from: https://stat.fi/en/luokitukset/ammatti/ammatti_1_20010101

[26] Statistics Norway. Standard for yrkesklassifisering (Standard Classification of Occupations) Oslo–Kongsvinger1998 [cited 20.06.2022]. Available from: https://www.ssb.no/a/publikasjoner/pdf/nos_c521/nos_c521.pdf

[27] Solovieva S, Undem K, Falkstedt D, Johansson G, Kristensen P, Pedersen J et al. Utilizing a Nordic Crosswalk for Occupational Coding in an Analysis on Occupation-Specific Prolonged Sickness Absence among 7 Million Employees in Denmark, Finland, Norway and Sweden. Int J Environ Res Public Health 2022 Nov;19(23):15674. 10.3390/ijerph192315674 36497749 PMC9737405

[28] Sullivan DF. A single index of mortality and morbidity. HSMHA Health Rep 1971 Apr;86(4):347–54. 10.2307/4594169 5554262 PMC1937122

[29] Dudel C. Healthy and unhealthy working-life expectancy: opportunities and challenges. Lancet Healthy Longev 2021 Oct;2(10):e604–5. 10.1016/S2666-7568(21)00211-7 36098011

[30] Juranek S, Paetzold J, Winner H, Zoutman F. Labor market effects of COVID-19 in Sweden and its neighbors: Evidence from novel administrative data. Discussion paper. SSRN Electronic Journal: NHH Dept. of Business and Management Science; 2020. Report No.: 8/2020. 10.2139/ssrn.3671259

[31] Junna L, Tarkiainen L, Leinonen T, Korhonen K, Martikainen P. Trends in working life expectancy by education and occupational social class in Finland, 1991–2020. Finnish Centre for Pensions; 2024. Report No.: 951691375X.

[32] Rugulies R, Framke E, Sørensen JK, Svane-Petersen AC, Alexanderson K, Bonde JP et al. Persistent and changing job strain and risk of coronary heart disease. A population-based cohort study of 1.6 million employees in Denmark. Scand J Work Environ Health 2020 Sep;46(5):498–507. 10.5271/sjweh.3891 32202306 PMC7737794

[33] Weber D, Loichinger E. Live longer, retire later? Developments of healthy life expectancies and working life expectancies between age 50-59 and age 60-69 in Europe. Eur J Ageing 2020 Dec;19(1):75–93. 10.1007/s10433-020-00592-5 35241999 PMC8881563

[34] Lievre A, Jusot F, Barnay T, Sermet C, Brouard N, Robine JM et al. Healthy working life expectancies at age 50 in Europe: a new indicator. J Nutr Health Aging 2007;11(6):508–14. 17985068

[35] Larsen M, Pedersen PJ. Pathways to early retirement in Denmark, 1984-2000. Int J Manpow 2008;29(5):384–409. 10.1108/01437720810888544

[36] Merkus SL, Hoff R, Hasting RL, Undem K, Robroek SJ, Gran JM et al. Gender and educational differences in work participation and working years lost in Norway. Scand J Work Environ Health 2024 Sep;50(6):426–36. 10.5271/sjweh.4166 38785113 PMC11391266

[37] Dudel C, López Gómez MA, Benavides FG, Myrskylä M. The Length of Working Life in Spain: Levels, Recent Trends, and the Impact of the Financial Crisis. Eur J Popul 2018 Jan;34(5):769–91. 10.1007/s10680-017-9458-9 30976261 PMC6261850

[38] Lorenti A, Dudel C, Myrskylä M. The legacy of the great recession in Italy: a wider geographical, gender, and generational gap in working life expectancy. Soc Indic Res 2019;142:283–303. 10.1007/s11205-018-1910-7

[39] Dudel C, Loichinger E, Klüsener S, Sulak H, Myrskylä M. The Extension of Late Working Life in Germany: Trends, Inequalities, and the East-West Divide. Demography 2023 Aug;60(4):1115–37. 10.1215/00703370-10850040 37395719

[40] Peters S, Undem K, Solovieva S, Selander J, Schlünssen V, Oude Hengel KM et al. Narrative review of occupational exposures and noncommunicable diseases. Ann Work Expo Health 2024 Jul;68(6):562–80. 10.1093/annweh/wxae045 38815981 PMC11229329

[41] Hansen CD, Andersen JH. Going ill to work--what personal circumstances, attitudes and work-related factors are associated with sickness presenteeism? Soc Sci Med 2008 Sep;67(6):956–64. 10.1016/j.socscimed.2008.05.022 18571821

[42] Ravesteijn B, Kippersluis HV, Doorslaer EV. The wear and tear on health: what is the role of occupation? Health Econ 2018 Feb;27(2):e69–86. 10.1002/hec.3563 28901590 PMC5849488

[43] Laaksonen M, Roos E, Rahkonen O, Martikainen P, Lahelma E. Influence of material and behavioural factors on occupational class differences in health. J Epidemiol Community Health 2005 Feb;59(2):163–9. 10.1136/jech.2003.019323 15650150 PMC1732992

