# Supplementary material for: Occupational differences in working life expectancy and working years lost in Nordic countries
Source: Scand J Work Environ Health. 2025 Aug 29;51(5):423–32. doi: 10.5271/sjweh.4239 (PMC12414609; doi:10.5271/sjweh.4239)
Supplement: Supplementary material [file SJWEH-51-423-S001.pdf]

Occupational differences in working life expectancy and working years lost in Nordic countries[1]

by Karina Undem, MPhil, [2] ***Taina Leinonen, PhD, Daniel Falkstedt, PhD, Gun Johansson, PhD, Jacob Pedersen, PhD,*** [5] ***Eira Viikari-Juntura, MD, PhD, Ingrid Sivesind Mehlum, MD, PhD, Svetlana Solovieva, PhD***

1. Supplementary material
2. Correspondence to: Karina Undem, National Institute of Occupational Health, Department of Occupational Medicine and Epidemiology, PB 5330 Majorstuen, 0304 Oslo, Norway. [E-mail: karina.undem@stami.no]

Supplementary table S1: Working life expectancy (WLE) and Working years lost (WYL) calculated at age 30, 35, 40, 45, 50 and 55 in the general employed population in Denmark, Finland and Norway

			WLE	WYL												
				Sickness absence (SA)			Time-restricted work disability	Unemployment	Disability retirement (DR)			Old-age retirement (OAR)				Other
				SA	Partial SA	total			DR	Partial DR	total	OAR	Partial OAR	VER[a]	total	
Men	Age 30	Denmark	26.33	0.85	NA	0.85	NA	2.24	2.34	NA	2.34	0.40	NA	1.11	1.51	0.66
		Finland	30.21	0.47	0.02	0.50	0.15	1.03	0.26	0.19	0.45	0.67	0.27	NA	0.94	0.52
		Norway	28.85	0.98	0.41	1.40	0.50	0.56	0.64	0.17	0.82	1.25	0.11	NA	1.36	1.00
	Age 35	Denmark	22.41	0.75	NA	0.75	NA	1.80	2.21	NA	2.21	0.40	NA	1.12	1.52	0.32
		Finland	25.68	0.44	0.02	0.46	0.13	0.85	0.26	0.19	0.45	0.67	0.27	NA	0.94	0.41
		Norway	24.47	0.87	0.38	1.24	0.45	0.45	0.61	0.17	0.79	1.25	0.11	NA	1.37	0.80
	Age 40	Denmark	18.30	0.64	NA	0.64	NA	1.44	2.03	NA	2.03	0.40	NA	1.12	1.52	0.16
		Finland	21.10	0.40	0.02	0.41	0.12	0.71	0.25	0.19	0.44	0.68	0.27	NA	0.94	0.32
		Norway	20.05	0.74	0.33	1.07	0.40	0.35	0.58	0.17	0.75	1.26	0.11	NA	1.37	0.62
	Age 45	Denmark	14.15	0.52	NA	0.52	NA	1.12	1.80	NA	1.80	0.41	NA	1.13	1.53	0.09
		Finland	16.54	0.35	0.02	0.36	0.10	0.57	0.24	0.19	0.43	0.68	0.27	NA	0.95	0.25
		Norway	15.65	0.61	0.27	0.88	0.34	0.27	0.53	0.16	0.69	1.26	0.11	NA	1.37	0.48
	Age 50	Denmark	10.11	0.40	NA	0.40	NA	0.80	1.50	NA	1.50	0.41	NA	1.14	1.55	0.05
		Finland	12.06	0.29	0.01	0.30	0.08	0.43	0.22	0.18	0.41	0.69	0.27	NA	0.96	0.19
		Norway	11.31	0.46	0.21	0.67	0.26	0.19	0.46	0.14	0.60	1.27	0.11	NA	1.38	0.35
	Age 55	Denmark	6.20	0.26	NA	0.26	NA	0.48	1.10	NA	1.10	0.42	NA	1.16	1.58	0.02
		Finland	7.64	0.21	0.01	0.22	0.05	0.29	0.20	0.16	0.36	0.68	0.28	NA	0.96	0.13
		Norway	7.02	0.31	0.14	0.44	0.18	0.12	0.36	0.11	0.47	1.28	0.11	NA	1.39	0.23
Women	Age 30	Denmark	23.70	1.35	NA	1.35	NA	2.34	3.23	NA	3.23	0.42	NA	1.61	2.03	1.68
		Finland	30.02	0.59	0.07	0.66	0.25	1.36	0.17	0.39	0.56	0.61	0.37	NA	0.98	0.59

	Norway	27.32	1.53	1.09	2.63	1.03	0.41	0.97	0.67	1.64	0.61	0.13	NA	0.74	0.93
Age 35	Denmark	20.52	1.16	NA	1.16	NA	1.83	3.09	NA	3.09	0.42	NA	1.61	2.03	0.74
	Finland	25.63	0.53	0.06	0.59	0.23	1.12	0.16	0.39	0.56	0.61	0.37	NA	0.98	0.37
	Norway	23.29	1.26	0.94	2.20	0.93	0.32	0.94	0.67	1.61	0.61	0.13	NA	0.74	0.65
Age 40	Denmark	16.92	0.94	NA	0.94	NA	1.40	2.86	NA	2.86	0.42	NA	1.61	2.03	0.28
	Finland	21.13	0.47	0.06	0.52	0.21	0.90	0.16	0.39	0.55	0.61	0.37	NA	0.98	0.25
	Norway	19.17	1.02	0.78	1.79	0.80	0.24	0.90	0.65	1.55	0.61	0.13	NA	0.74	0.46
Age 45	Denmark	13.08	0.73	NA	0.73	NA	1.03	2.52	NA	2.52	0.42	NA	1.62	2.04	0.12
	Finland	16.60	0.39	0.05	0.44	0.17	0.70	0.15	0.39	0.54	0.61	0.37	NA	0.98	0.18
	Norway	15.04	0.80	0.62	1.42	0.64	0.18	0.83	0.60	1.44	0.61	0.13	NA	0.75	0.34
Age 50	Denmark	9.23	0.52	NA	0.52	NA	0.70	2.06	NA	2.06	0.42	NA	1.63	2.05	0.06
	Finland	12.10	0.31	0.04	0.35	0.13	0.51	0.14	0.37	0.51	0.61	0.38	NA	0.99	0.13
	Norway	10.96	0.60	0.46	1.05	0.48	0.12	0.72	0.53	1.25	0.61	0.14	NA	0.75	0.24
Age 55	Denmark	5.50	0.30	NA	0.30	NA	0.40	1.48	NA	1.48	0.43	NA	1.65	2.08	0.02
	Finland	7.64	0.21	0.02	0.24	0.08	0.32	0.12	0.34	0.46	0.62	0.38	NA	1.00	0.09
	Norway	6.96	0.39	0.29	0.69	0.31	0.07	0.56	0.41	0.97	0.62	0.14	NA	0.75	0.16

a. VER = Voluntary early retirement

Supplementary table S2: Working life expectancy (WLE) and Working years lost (WYL) calculated at age 30, 35, 40, 45, 50 and 55 among men, by major occupational group and country

			WLE	WYL												
				Sickness absence (SA)			Time-restricted work disability	Unemployment	Disability retirement (DR)			Old-age retirement (OAR)				Other
**Age**	**Country**	**Occupational group**		*SA*	*Partial SA*	*Total*			*DR*	*Partial DR*	*Total*	*OAR*	*Partial OAR*	VER[a]	*Total*	
Age 30	Denmark	Legislators, senior officials and managers	32.64	0.34	NA	0.34	NA	0.11	0.15	NA	0.15	0.92	NA	0.60	1.52	0.20
		Professionals	31.91	0.44	NA	0.44	NA	0.19	0.41	NA	0.41	0.84	NA	0.77	1.60	0.41
		Technicians and associate professionals	31.37	0.47	NA	0.47	NA	0.23	0.99	NA	0.99	0.79	NA	0.78	1.57	0.28
		Clerks	31.18	0.54	NA	0.54	NA	0.36	0.90	NA	0.90	0.68	NA	0.87	1.55	0.43
		Service and sales workers	29.80	0.72	NA	0.72	NA	0.57	1.57	NA	1.57	0.50	NA	1.15	1.65	0.61
		Skilled agricultural and fishery workers	30.54	0.67	NA	0.67	NA	0.74	0.99	NA	0.99	0.77	NA	0.99	1.76	0.30
		Craft and related trades workers	30.91	0.82	NA	0.82	NA	0.48	0.72	NA	0.72	0.57	NA	1.14	1.71	0.28
		Plant and machine operators and assemblers	31.22	0.71	NA	0.71	NA	0.38	0.65	NA	0.65	0.61	NA	1.00	1.61	0.32
		Elementary occupations	29.09	0.75	NA	0.75	NA	1.00	2.01	NA	2.01	0.53	NA	1.08	1.61	0.41
	Finland	Legislators, senior officials and managers	32.73	0.31	0.02	0.33	0.06	0.30	0.08	0.08	0.15	0.62	0.23	NA	0.85	0.54
		Professionals	31.66	0.24	0.02	0.26	0.06	0.75	0.12	0.13	0.25	0.57	0.43	NA	1.00	0.47
		Technicians and associate professionals	31.25	0.35	0.05	0.39	0.10	0.72	0.18	0.16	0.34	0.69	0.32	NA	1.01	0.46
		Clerks	29.76	0.49	0.16	0.65	0.24	1.27	0.29	0.40	0.69	0.68	0.32	NA	1.01	0.58
		Service and sales workers	28.96	0.57	0.08	0.65	0.22	1.57	0.50	0.23	0.73	0.94	0.20	NA	1.14	0.63
		Skilled agricultural and fishery workers	30.21	0.55	0.14	0.70	0.19	0.71	0.40	0.28	0.69	0.58	0.13	NA	0.71	0.60
		Craft and related trades workers	29.43	0.67	0.05	0.71	0.21	1.42	0.29	0.24	0.53	0.70	0.23	NA	0.93	0.59
		Plant and machine operators and assemblers	29.93	0.68	0.06	0.74	0.19	1.03	0.35	0.21	0.56	0.77	0.22	NA	1.00	0.47
		Elementary occupations	27.64	0.68	0.15	0.83	0.24	2.36	0.59	0.27	0.86	0.73	0.21	NA	0.93	0.58
	Norway	Legislators, senior officials and managers	30.43	0.68	0.36	1.04	0.27	0.24	0.14	0.07	0.21	1.21	0.11	NA	1.32	1.11
		Professionals	31.28	0.49	0.38	0.87	0.22	0.22	0.14	0.11	0.26	0.97	0.17	NA	1.14	0.61
		Technicians and associate professionals	30.15	0.75	0.39	1.14	0.31	0.43	0.23	0.15	0.37	1.34	0.13	NA	1.47	0.72
		Clerks	28.34	1.07	0.52	1.59	0.56	0.66	0.68	0.29	0.96	1.57	0.11	NA	1.68	0.67
		Service and sales workers	27.62	1.29	0.50	1.80	0.84	0.61	0.98	0.38	1.35	1.20	0.07	NA	1.27	0.95
		Skilled agricultural and fishery workers	26.89	1.15	0.38	1.53	0.64	0.60	1.26	0.23	1.49	1.06	0.12	NA	1.18	1.82
		Craft and related trades workers	27.77	1.42	0.51	1.93	0.70	0.77	0.38	0.20	0.58	1.54	0.09	NA	1.63	1.13
		Plant and machine operators and assemblers	27.45	1.57	0.41	1.98	0.66	0.72	0.63	0.19	0.82	1.58	0.07	NA	1.65	0.96
		Elementary occupations	26.96	1.53	0.48	2.01	0.48	1.21	1.16	0.31	1.47	1.27	0.06	NA	1.33	1.22
Age 35	Denmark	Legislators, senior officials and managers	27.80	0.29	NA	0.29	NA	0.09	0.14	NA	0.14	0.92	NA	0.60	1.52	0.10
		Professionals	27.21	0.40	NA	0.40	NA	0.15	0.40	NA	0.40	0.84	NA	0.77	1.60	0.20
		Technicians and associate professionals	26.67	0.42	NA	0.42	NA	0.19	0.93	NA	0.93	0.80	NA	0.78	1.57	0.13
		Clerks	26.64	0.49	NA	0.49	NA	0.28	0.85	NA	0.85	0.68	NA	0.87	1.55	0.18
		Service and sales workers	25.42	0.64	NA	0.64	NA	0.45	1.46	NA	1.46	0.50	NA	1.15	1.65	0.29
		Skilled agricultural and fishery workers	25.91	0.61	NA	0.61	NA	0.65	0.91	NA	0.91	0.77	NA	0.99	1.76	0.16
		Craft and related trades workers	26.29	0.71	NA	0.71	NA	0.39	0.69	NA	0.69	0.57	NA	1.14	1.71	0.13
		Plant and machine operators and assemblers	26.56	0.62	NA	0.62	NA	0.30	0.61	NA	0.61	0.61	NA	1.00	1.61	0.18
		Elementary occupations	24.71	0.67	NA	0.67	NA	0.82	1.85	NA	1.85	0.53	NA	1.08	1.61	0.22
	Finland	Legislators, senior officials and managers	27.93	0.27	0.01	0.29	0.05	0.25	0.07	0.08	0.15	0.62	0.23	NA	0.85	0.43

		Professionals	27.00	0.22	0.02	0.24	0.06	0.61	0.12	0.13	0.25	0.57	0.43	NA	1.00	0.35
		Technicians and associate professionals	26.57	0.32	0.03	0.35	0.09	0.62	0.18	0.16	0.33	0.69	0.32	NA	1.01	0.36
		Clerks	25.34	0.46	0.11	0.57	0.23	1.05	0.29	0.40	0.68	0.69	0.32	NA	1.01	0.47
		Service and sales workers	24.45	0.53	0.04	0.57	0.21	1.32	0.49	0.23	0.72	0.95	0.20	NA	1.14	0.52
		Skilled agricultural and fishery workers	25.63	0.52	0.08	0.60	0.18	0.57	0.39	0.28	0.67	0.58	0.13	NA	0.71	0.52
		Craft and related trades workers	24.98	0.61	0.02	0.64	0.19	1.20	0.28	0.24	0.52	0.70	0.23	NA	0.93	0.50
		Plant and machine operators and assemblers	25.35	0.63	0.05	0.67	0.17	0.88	0.34	0.21	0.56	0.78	0.22	NA	1.00	0.39
		Elementary occupations	23.40	0.63	0.06	0.68	0.23	2.02	0.57	0.27	0.84	0.73	0.21	NA	0.94	0.52
	Norway	Legislators, senior officials and managers	25.91	0.59	0.32	0.91	0.25	0.19	0.14	0.07	0.21	1.21	0.11	NA	1.32	0.88
		Professionals	26.57	0.45	0.35	0.80	0.20	0.18	0.14	0.11	0.25	0.97	0.17	NA	1.15	0.48
		Technicians and associate professionals	25.53	0.68	0.36	1.04	0.29	0.35	0.22	0.14	0.37	1.35	0.13	NA	1.47	0.59
		Clerks	24.02	0.95	0.47	1.42	0.50	0.52	0.66	0.28	0.94	1.58	0.11	NA	1.68	0.50
		Service and sales workers	23.32	1.15	0.46	1.62	0.76	0.49	0.95	0.37	1.33	1.20	0.07	NA	1.28	0.72
		Skilled agricultural and fishery workers	22.92	1.04	0.36	1.40	0.57	0.49	1.20	0.23	1.42	1.06	0.12	NA	1.19	1.46
		Craft and related trades workers	23.43	1.25	0.46	1.71	0.64	0.65	0.38	0.20	0.58	1.54	0.09	NA	1.63	0.93
		Plant and machine operators and assemblers	23.24	1.38	0.37	1.75	0.59	0.56	0.61	0.19	0.80	1.58	0.07	NA	1.65	0.76
		Elementary occupations	22.91	1.32	0.44	1.76	0.44	0.95	1.10	0.31	1.41	1.27	0.06	NA	1.33	0.93
Age 40	Denmark	Legislators, senior officials and managers	22.92	0.25	NA	0.25	NA	0.08	0.13	NA	0.13	0.92	NA	0.60	1.52	0.05
		Professionals	22.43	0.35	NA	0.35	NA	0.12	0.38	NA	0.38	0.84	NA	0.77	1.60	0.09
		Technicians and associate professionals	21.92	0.36	NA	0.36	NA	0.16	0.86	NA	0.86	0.80	NA	0.78	1.57	0.06
		Clerks	21.93	0.41	NA	0.41	NA	0.22	0.80	NA	0.80	0.68	NA	0.87	1.55	0.08
		Service and sales workers	20.90	0.55	NA	0.55	NA	0.36	1.31	NA	1.31	0.50	NA	1.15	1.65	0.15
		Skilled agricultural and fishery workers	21.29	0.53	NA	0.53	NA	0.53	0.78	NA	0.78	0.77	NA	0.99	1.76	0.11
		Craft and related trades workers	21.61	0.60	NA	0.60	NA	0.33	0.64	NA	0.64	0.57	NA	1.14	1.72	0.06
		Plant and machine operators and assemblers	21.85	0.53	NA	0.53	NA	0.23	0.56	NA	0.56	0.62	NA	1.00	1.61	0.12
		Elementary occupations	20.31	0.56	NA	0.56	NA	0.65	1.64	NA	1.64	0.53	NA	1.08	1.61	0.12
	Finland	Legislators, senior officials and managers	23.08	0.25	0.01	0.26	0.04	0.22	0.07	0.08	0.15	0.62	0.23	NA	0.85	0.35
		Professionals	22.26	0.20	0.02	0.22	0.06	0.51	0.12	0.13	0.24	0.57	0.43	NA	1.00	0.27
		Technicians and associate professionals	21.84	0.29	0.03	0.32	0.08	0.53	0.17	0.16	0.33	0.69	0.32	NA	1.01	0.29
		Clerks	20.75	0.41	0.09	0.50	0.19	0.87	0.28	0.39	0.67	0.69	0.32	NA	1.01	0.35
		Service and sales workers	20.07	0.48	0.04	0.51	0.19	1.09	0.47	0.23	0.70	0.95	0.20	NA	1.15	0.40
		Skilled agricultural and fishery workers	21.18	0.47	0.08	0.55	0.16	0.43	0.37	0.28	0.65	0.58	0.13	NA	0.72	0.39
		Craft and related trades workers	20.51	0.55	0.02	0.57	0.17	1.01	0.27	0.24	0.52	0.71	0.23	NA	0.94	0.39
		Plant and machine operators and assemblers	20.79	0.56	0.05	0.60	0.15	0.74	0.34	0.22	0.55	0.78	0.23	NA	1.01	0.30
		Elementary occupations	19.18	0.55	0.03	0.58	0.20	1.69	0.53	0.27	0.81	0.74	0.21	NA	0.95	0.40
	Norway	Legislators, senior officials and managers	21.30	0.50	0.28	0.78	0.23	0.15	0.14	0.07	0.20	1.21	0.11	NA	1.32	0.69
		Professionals	21.85	0.40	0.31	0.71	0.19	0.15	0.14	0.11	0.25	0.97	0.17	NA	1.15	0.37
		Technicians and associate professionals	20.93	0.59	0.31	0.90	0.26	0.29	0.22	0.14	0.36	1.35	0.13	NA	1.47	0.47
		Clerks	19.66	0.81	0.40	1.21	0.43	0.39	0.63	0.27	0.90	1.58	0.11	NA	1.69	0.37
		Service and sales workers	19.04	0.99	0.40	1.39	0.67	0.38	0.90	0.36	1.26	1.21	0.07	NA	1.28	0.56
		Skilled agricultural and fishery workers	18.65	0.92	0.31	1.23	0.49	0.39	1.13	0.23	1.35	1.06	0.12	NA	1.19	1.15
		Craft and related trades workers	19.14	1.06	0.40	1.46	0.58	0.53	0.37	0.20	0.57	1.54	0.09	NA	1.63	0.72
		Plant and machine operators and assemblers	18.96	1.16	0.32	1.48	0.52	0.43	0.59	0.19	0.78	1.59	0.07	NA	1.65	0.58

		Elementary occupations	18.82	1.12	0.38	1.50	0.38	0.74	1.03	0.30	1.33	1.27	0.06	NA	1.33	0.68
Age 45	Denmark	Legislators, senior officials and managers	18.02	0.21	NA	0.21	NA	0.06	0.13	NA	0.13	0.92	NA	0.60	1.53	0.02
		Professionals	17.60	0.29	NA	0.29	NA	0.10	0.35	NA	0.35	0.84	NA	0.77	1.60	0.04
		Technicians and associate professionals	17.13	0.30	NA	0.30	NA	0.13	0.77	NA	0.77	0.80	NA	0.78	1.57	0.03
		Clerks	17.19	0.34	NA	0.34	NA	0.17	0.69	NA	0.69	0.68	NA	0.87	1.55	0.04
		Service and sales workers	16.32	0.45	NA	0.45	NA	0.28	1.15	NA	1.15	0.50	NA	1.15	1.65	0.09
		Skilled agricultural and fishery workers	16.70	0.43	NA	0.43	NA	0.43	0.62	NA	0.62	0.77	NA	0.99	1.76	0.06
		Craft and related trades workers	16.87	0.49	NA	0.49	NA	0.26	0.59	NA	0.59	0.58	NA	1.14	1.72	0.03
		Plant and machine operators and assemblers	17.11	0.44	NA	0.44	NA	0.18	0.51	NA	0.51	0.62	NA	1.00	1.61	0.07
		Elementary occupations	15.85	0.45	NA	0.45	NA	0.50	1.43	NA	1.43	0.53	NA	1.08	1.61	0.07
	Finland	Legislators, senior officials and managers	18.25	0.21	0.01	0.22	0.04	0.19	0.07	0.07	0.14	0.62	0.23	NA	0.85	0.27
		Professionals	17.54	0.18	0.01	0.19	0.05	0.42	0.11	0.13	0.24	0.57	0.43	NA	1.00	0.21
		Technicians and associate professionals	17.13	0.26	0.02	0.28	0.07	0.44	0.17	0.15	0.32	0.70	0.32	NA	1.02	0.23
		Clerks	16.21	0.35	0.07	0.42	0.16	0.67	0.26	0.38	0.64	0.69	0.33	NA	1.01	0.27
		Service and sales workers	15.61	0.40	0.03	0.43	0.16	0.86	0.44	0.22	0.66	0.96	0.20	NA	1.16	0.31
		Skilled agricultural and fishery workers	16.74	0.41	0.07	0.49	0.14	0.34	0.35	0.28	0.63	0.59	0.13	NA	0.73	0.31
		Craft and related trades workers	16.08	0.48	0.02	0.50	0.14	0.81	0.26	0.24	0.50	0.71	0.23	NA	0.95	0.30
		Plant and machine operators and assemblers	16.23	0.49	0.05	0.53	0.13	0.60	0.33	0.21	0.54	0.79	0.23	NA	1.01	0.23
		Elementary occupations	14.93	0.47	0.03	0.50	0.16	1.35	0.49	0.27	0.76	0.74	0.21	NA	0.95	0.29
	Norway	Legislators, senior officials and managers	16.71	0.41	0.23	0.64	0.20	0.12	0.13	0.06	0.20	1.22	0.11	NA	1.33	0.54
		Professionals	17.18	0.34	0.26	0.60	0.16	0.12	0.13	0.11	0.23	0.98	0.17	NA	1.15	0.29
		Technicians and associate professionals	16.35	0.49	0.26	0.75	0.23	0.22	0.21	0.13	0.34	1.35	0.13	NA	1.48	0.37
		Clerks	15.35	0.64	0.33	0.96	0.35	0.29	0.58	0.26	0.84	1.59	0.11	NA	1.69	0.26
		Service and sales workers	14.77	0.81	0.34	1.14	0.56	0.29	0.81	0.34	1.15	1.21	0.07	NA	1.28	0.43
		Skilled agricultural and fishery workers	14.54	0.77	0.24	1.01	0.41	0.28	1.00	0.22	1.23	1.07	0.12	NA	1.19	0.91
		Craft and related trades workers	14.86	0.86	0.33	1.19	0.50	0.40	0.35	0.19	0.54	1.55	0.09	NA	1.64	0.54
		Plant and machine operators and assemblers	14.74	0.94	0.25	1.19	0.44	0.33	0.55	0.17	0.73	1.60	0.07	NA	1.66	0.43
		Elementary occupations	14.71	0.90	0.31	1.21	0.31	0.55	0.94	0.28	1.22	1.27	0.06	NA	1.33	0.47
Age 50	Denmark	Legislators, senior officials and managers	13.11	0.17	NA	0.17	NA	0.04	0.12	NA	0.12	0.93	NA	0.60	1.53	0.01
		Professionals	12.75	0.23	NA	0.23	NA	0.07	0.30	NA	0.30	0.84	NA	0.77	1.60	0.02
		Technicians and associate professionals	12.37	0.24	NA	0.24	NA	0.09	0.64	NA	0.64	0.80	NA	0.78	1.57	0.02
		Clerks	12.43	0.27	NA	0.27	NA	0.13	0.59	NA	0.59	0.68	NA	0.87	1.55	0.02
		Service and sales workers	11.75	0.35	NA	0.35	NA	0.19	0.95	NA	0.95	0.50	NA	1.15	1.65	0.04
		Skilled agricultural and fishery workers	12.09	0.33	NA	0.33	NA	0.32	0.47	NA	0.47	0.77	NA	0.99	1.76	0.03
		Craft and related trades workers	12.16	0.37	NA	0.37	NA	0.20	0.50	NA	0.50	0.58	NA	1.14	1.72	0.01
		Plant and machine operators and assemblers	12.40	0.33	NA	0.33	NA	0.13	0.43	NA	0.43	0.62	NA	1.00	1.62	0.04
		Elementary occupations	11.37	0.34	NA	0.34	NA	0.35	1.19	NA	1.19	0.53	NA	1.08	1.61	0.04
	Finland	Legislators, senior officials and managers	13.43	0.18	0.01	0.19	0.03	0.15	0.06	0.07	0.14	0.62	0.23	NA	0.85	0.20
		Professionals	12.85	0.15	0.01	0.16	0.04	0.31	0.10	0.12	0.23	0.57	0.44	NA	1.01	0.15
		Technicians and associate professionals	12.53	0.21	0.02	0.23	0.05	0.34	0.15	0.15	0.30	0.70	0.32	NA	1.02	0.17
		Clerks	11.78	0.29	0.06	0.35	0.11	0.48	0.23	0.37	0.60	0.69	0.33	NA	1.01	0.19
		Service and sales workers	11.37	0.33	0.03	0.36	0.12	0.63	0.41	0.21	0.62	0.97	0.20	NA	1.18	0.23
		Skilled agricultural and fishery workers	12.23	0.34	0.08	0.42	0.11	0.25	0.32	0.26	0.58	0.60	0.14	NA	0.73	0.23

		Craft and related trades workers	11.66	0.40	0.02	0.42	0.11	0.61	0.25	0.24	0.49	0.72	0.24	NA	0.96	0.22
		Plant and machine operators and assemblers	11.78	0.40	0.04	0.44	0.10	0.45	0.32	0.20	0.52	0.80	0.23	NA	1.03	0.17
		Elementary occupations	10.92	0.39	0.02	0.40	0.12	1.00	0.43	0.27	0.70	0.76	0.22	NA	0.97	0.21
	Norway	Legislators, senior officials and managers	12.14	0.31	0.18	0.49	0.16	0.09	0.12	0.06	0.18	1.22	0.11	NA	1.33	0.40
		Professionals	12.53	0.27	0.20	0.47	0.13	0.09	0.12	0.10	0.21	0.98	0.17	NA	1.16	0.21
		Technicians and associate professionals	11.82	0.38	0.20	0.57	0.18	0.16	0.19	0.12	0.31	1.36	0.13	NA	1.48	0.28
		Clerks	10.98	0.49	0.24	0.72	0.27	0.20	0.50	0.23	0.73	1.59	0.11	NA	1.70	0.17
		Service and sales workers	10.63	0.61	0.26	0.87	0.43	0.20	0.71	0.30	1.01	1.22	0.07	NA	1.29	0.33
		Skilled agricultural and fishery workers	10.44	0.59	0.19	0.79	0.30	0.19	0.89	0.19	1.07	1.08	0.12	NA	1.20	0.72
		Craft and related trades workers	10.65	0.66	0.25	0.91	0.40	0.29	0.32	0.17	0.49	1.55	0.09	NA	1.65	0.38
		Plant and machine operators and assemblers	10.57	0.69	0.19	0.88	0.34	0.23	0.49	0.15	0.64	1.61	0.07	NA	1.68	0.30
		Elementary occupations	10.58	0.68	0.24	0.93	0.24	0.39	0.80	0.25	1.05	1.27	0.06	NA	1.33	0.33
Age 55	Denmark	Legislators, senior officials and managers	8.20	0.12	NA	0.12	NA	0.03	0.10	NA	0.10	0.93	NA	0.60	1.53	0.01
		Professionals	7.94	0.16	NA	0.16	NA	0.04	0.23	NA	0.23	0.84	NA	0.77	1.61	0.01
		Technicians and associate professionals	7.67	0.17	NA	0.17	NA	0.06	0.47	NA	0.47	0.80	NA	0.78	1.58	0.01
		Clerks	7.70	0.19	NA	0.19	NA	0.07	0.47	NA	0.47	0.68	NA	0.87	1.55	0.00
		Service and sales workers	7.25	0.23	NA	0.23	NA	0.11	0.70	NA	0.70	0.50	NA	1.15	1.66	0.02
		Skilled agricultural and fishery workers	7.51	0.22	NA	0.22	NA	0.20	0.29	NA	0.29	0.77	NA	0.99	1.76	0.01
		Craft and related trades workers	7.51	0.24	NA	0.24	NA	0.12	0.39	NA	0.39	0.58	NA	1.14	1.72	0.01
		Plant and machine operators and assemblers	7.70	0.22	NA	0.22	NA	0.08	0.34	NA	0.34	0.62	NA	1.00	1.62	0.02
		Elementary occupations	6.98	0.22	NA	0.22	NA	0.20	0.91	NA	0.91	0.53	NA	1.09	1.62	0.02
	Finland	Legislators, senior officials and managers	8.60	0.13	0.01	0.14	0.02	0.11	0.06	0.06	0.12	0.62	0.23	NA	0.85	0.14
		Professionals	8.19	0.11	0.01	0.11	0.02	0.20	0.09	0.11	0.20	0.57	0.44	NA	1.01	0.10
		Technicians and associate professionals	7.95	0.15	0.02	0.17	0.03	0.23	0.13	0.13	0.26	0.69	0.33	NA	1.02	0.12
		Clerks	7.45	0.20	0.06	0.25	0.07	0.32	0.20	0.32	0.51	0.68	0.33	NA	1.02	0.14
		Service and sales workers	7.06	0.23	0.02	0.25	0.07	0.41	0.37	0.19	0.55	0.98	0.21	NA	1.18	0.15
		Skilled agricultural and fishery workers	7.81	0.24	0.07	0.31	0.06	0.16	0.29	0.21	0.50	0.61	0.14	NA	0.74	0.18
		Craft and related trades workers	7.31	0.29	0.02	0.31	0.07	0.41	0.23	0.21	0.44	0.73	0.24	NA	0.97	0.14
		Plant and machine operators and assemblers	7.36	0.28	0.04	0.32	0.06	0.31	0.29	0.19	0.48	0.80	0.23	NA	1.04	0.12
		Elementary occupations	6.82	0.27	0.02	0.29	0.07	0.66	0.37	0.24	0.61	0.77	0.22	NA	0.99	0.13
	Norway	Legislators, senior officials and managers	7.61	0.22	0.12	0.34	0.12	0.05	0.10	0.05	0.14	1.23	0.11	NA	1.34	0.26
		Professionals	7.94	0.19	0.14	0.33	0.09	0.06	0.10	0.08	0.18	0.99	0.17	NA	1.16	0.14
		Technicians and associate professionals	7.34	0.26	0.13	0.39	0.13	0.10	0.15	0.10	0.25	1.37	0.13	NA	1.50	0.19
		Clerks	6.69	0.32	0.15	0.47	0.19	0.13	0.40	0.19	0.58	1.60	0.11	NA	1.71	0.11
		Service and sales workers	6.55	0.40	0.17	0.58	0.29	0.12	0.55	0.23	0.77	1.23	0.08	NA	1.30	0.24
		Skilled agricultural and fishery workers	6.44	0.39	0.13	0.52	0.19	0.12	0.73	0.15	0.88	1.09	0.13	NA	1.22	0.47
		Craft and related trades workers	6.49	0.44	0.17	0.61	0.29	0.18	0.26	0.14	0.40	1.57	0.09	NA	1.66	0.23
		Plant and machine operators and assemblers	6.44	0.45	0.12	0.57	0.23	0.14	0.41	0.12	0.53	1.63	0.07	NA	1.70	0.19
		Elementary occupations	6.54	0.45	0.16	0.60	0.16	0.24	0.62	0.19	0.81	1.27	0.06	NA	1.33	0.21

a. VER = Voluntary early retirement

Supplementary table S3: Working life expectancy (WLE) and Working years lost (WYL) calculated at age 30, 35, 40, 45, 50 and 55 among women, by major occupational group and country

			WLE	WYL												
				Sickness absence (SA)			Time-restricted work disability	Unemployment	Disability retirement (DR)			Old-age retirement (OAR)				Other
**Age**	**Country**	**Occupational group**		*SA*	*Partial SA*	*Total*			*DR*	*Partial DR*	*Total*	*OAR*	*Partial OAR*	VER[a]	*Total*	
Age 30	Denmark	Legislators, senior officials and managers	31.23	0.73	NA	0.73	NA	0.16	0.36	NA	0.36	0.87	NA	0.55	1.42	1.10
		Professionals	29.78	1.05	NA	1.05	NA	0.28	0.80	NA	0.80	0.61	NA	1.13	1.74	1.29
		Technicians and associate professionals	30.26	0.85	NA	0.85	NA	0.22	0.72	NA	0.72	0.67	NA	1.10	1.77	1.15
		Clerks	30.00	0.88	NA	0.88	NA	0.40	0.83	NA	0.83	0.63	NA	1.08	1.71	1.13
		Service and sales workers	28.05	1.20	NA	1.20	NA	0.79	2.00	NA	2.00	0.55	NA	1.20	1.75	1.11
		Skilled agricultural and fishery workers	29.11	1.26	NA	1.26	NA	1.35	1.19	NA	1.19	0.53	NA	0.08	0.60	1.05
		Craft and related trades workers	28.81	1.29	NA	1.29	NA	0.62	1.02	NA	1.02	0.41	NA	1.66	2.07	1.18
		Plant and machine operators and assemblers	29.98	1.15	NA	1.15	NA	0.59	0.98	NA	0.98	0.54	NA	0.94	1.48	0.81
		Elementary occupations	26.51	1.20	NA	1.20	NA	1.24	3.08	NA	3.08	0.53	NA	1.16	1.69	1.14
	Finland	Legislators, senior officials and managers	32.53	0.40	0.04	0.44	0.08	0.39	0.06	0.10	0.15	0.45	0.19	NA	0.65	0.62
		Professionals	31.28	0.36	0.04	0.41	0.13	0.94	0.10	0.22	0.32	0.55	0.45	NA	1.00	0.59
		Technicians and associate professionals	31.05	0.52	0.08	0.59	0.22	0.77	0.11	0.38	0.49	0.61	0.42	NA	1.03	0.48
		Clerks	30.06	0.51	0.07	0.58	0.23	1.48	0.14	0.40	0.54	0.57	0.43	NA	1.00	0.52
		Service and sales workers	29.10	0.78	0.09	0.87	0.37	1.79	0.21	0.52	0.73	0.68	0.36	NA	1.03	0.64
		Skilled agricultural and fishery workers	29.67	0.80	0.03	0.83	0.38	1.18	0.33	0.41	0.74	0.61	0.17	NA	0.79	0.83
		Craft and related trades workers	28.92	0.74	0.06	0.80	0.27	2.08	0.27	0.31	0.58	0.70	0.24	NA	0.94	0.85
		Plant and machine operators and assemblers	29.63	0.79	0.08	0.87	0.29	1.73	0.21	0.38	0.59	0.71	0.34	NA	1.05	0.56
		Elementary occupations	26.96	0.91	0.10	1.01	0.43	3.24	0.35	0.61	0.97	0.62	0.28	NA	0.90	0.68
	Norway	Legislators, senior officials and managers	29.73	1.23	1.00	2.24	0.50	0.24	0.16	0.17	0.16	0.77	0.09	NA	0.86	0.92
		Professionals	29.53	1.15	1.11	2.26	0.60	0.20	0.31	0.43	0.74	0.66	0.22	NA	0.88	0.56
		Technicians and associate professionals	28.21	1.47	1.25	2.73	0.80	0.29	0.46	0.63	1.09	0.80	0.16	NA	0.95	0.63
		Clerks	26.93	1.32	1.00	2.32	1.07	0.57	0.98	0.84	1.82	0.82	0.15	NA	0.97	0.97
		Service and sales workers	25.97	1.99	1.15	3.14	1.48	0.47	1.24	1.03	2.26	0.41	0.08	NA	0.48	0.86
		Skilled agricultural and fishery workers	24.73	1.66	0.73	2.39	1.59	0.56	1.61	0.55	2.17	0.38	0.15	NA	0.53	2.87
		Craft and related trades workers	26.37	1.50	0.93	2.43	1.25	0.86	0.65	0.67	1.32	0.77	0.14	NA	0.91	1.20
		Plant and machine operators and assemblers	25.30	2.12	0.97	3.09	1.39	1.02	0.93	0.77	1.71	0.93	0.16	NA	1.09	1.12
		Elementary occupations	25.14	2.19	0.90	3.09	1.42	0.90	1.40	0.65	2.06	0.42	0.10	NA	0.51	1.39
Age 35	Denmark	Legislators, senior officials and managers	27.13	0.62	NA	0.62	NA	0.12	0.34	NA	0.34	0.87	NA	0.55	1.42	0.37
		Professionals	25.80	0.92	NA	0.92	NA	0.22	0.78	NA	0.78	0.61	NA	1.13	1.74	0.48
		Technicians and associate professionals	26.22	0.74	NA	0.74	NA	0.16	0.69	NA	0.69	0.67	NA	1.10	1.77	0.38
		Clerks	26.01	0.76	NA	0.76	NA	0.31	0.78	NA	0.78	0.63	NA	1.08	1.71	0.38
		Service and sales workers	24.22	1.03	NA	1.03	NA	0.61	1.87	NA	1.87	0.55	NA	1.20	1.75	0.45
		Skilled agricultural and fishery workers	25.17	1.07	NA	1.07	NA	1.21	1.09	NA	1.09	0.53	NA	0.08	0.60	0.41
		Craft and related trades workers	24.94	1.11	NA	1.11	NA	0.50	0.94	NA	0.94	0.41	NA	1.66	2.07	0.44
		Plant and machine operators and assemblers	25.75	0.99	NA	0.99	NA	0.49	0.93	NA	0.93	0.54	NA	0.94	1.48	0.36
		Elementary occupations	22.83	1.04	NA	1.04	NA	0.99	2.87	NA	2.87	0.53	NA	1.16	1.69	0.47
	Finland	Legislators, senior officials and managers	27.87	0.34	0.03	0.37	0.07	0.34	0.06	0.10	0.15	0.45	0.19	NA	0.65	0.41
		Professionals	26.80	0.33	0.04	0.37	0.12	0.73	0.10	0.22	0.32	0.55	0.45	NA	1.00	0.35

		Technicians and associate professionals	26.49	0.46	0.07	0.53	0.21	0.64	0.11	0.38	0.49	0.61	0.42	NA	1.03	0.31
		Clerks	25.62	0.46	0.06	0.53	0.21	1.24	0.14	0.40	0.54	0.57	0.43	NA	1.00	0.34
		Service and sales workers	24.81	0.69	0.08	0.77	0.34	1.50	0.21	0.52	0.73	0.68	0.36	NA	1.04	0.40
		Skilled agricultural and fishery workers	25.30	0.72	0.03	0.75	0.35	0.93	0.32	0.41	0.73	0.61	0.17	NA	0.79	0.63
		Craft and related trades workers	24.71	0.66	0.05	0.71	0.24	1.67	0.26	0.31	0.57	0.70	0.24	NA	0.94	0.60
		Plant and machine operators and assemblers	25.21	0.71	0.07	0.78	0.26	1.44	0.21	0.38	0.59	0.71	0.34	NA	1.05	0.39
		Elementary occupations	23.01	0.82	0.09	0.91	0.39	2.70	0.34	0.61	0.95	0.62	0.28	NA	0.90	0.44
	Norway	Legislators, senior officials and managers	25.59	0.96	0.84	1.79	0.45	0.18	0.16	0.16	0.16	0.77	0.09	NA	0.86	0.63
		Professionals	25.17	0.95	0.94	1.90	0.56	0.15	0.31	0.43	0.73	0.66	0.22	NA	0.88	0.37
		Technicians and associate professionals	24.04	1.21	1.07	2.28	0.73	0.24	0.46	0.62	1.08	0.80	0.16	NA	0.95	0.43
		Clerks	23.01	1.06	0.86	1.92	0.95	0.43	0.95	0.83	1.79	0.82	0.15	NA	0.98	0.63
		Service and sales workers	22.11	1.66	1.00	2.66	1.32	0.35	1.21	1.01	2.23	0.41	0.08	NA	0.49	0.57
		Skilled agricultural and fishery workers	21.19	1.30	0.64	1.94	1.42	0.46	1.53	0.54	2.07	0.38	0.15	NA	0.53	2.23
		Craft and related trades workers	22.50	1.21	0.79	2.00	1.08	0.67	0.63	0.66	1.29	0.77	0.14	NA	0.91	0.89
		Plant and machine operators and assemblers	21.48	1.78	0.83	2.60	1.27	0.82	0.91	0.77	1.68	0.93	0.16	NA	1.09	0.78
		Elementary occupations	21.49	1.83	0.80	2.62	1.30	0.71	1.37	0.65	2.02	0.42	0.10	NA	0.51	0.91
Age 40	Denmark	Legislators, senior officials and managers	22.59	0.51	NA	0.51	NA	0.09	0.32	NA	0.32	0.87	NA	0.55	1.42	0.07
		Professionals	21.42	0.76	NA	0.76	NA	0.17	0.73	NA	0.73	0.61	NA	1.13	1.74	0.13
		Technicians and associate professionals	21.77	0.61	NA	0.61	NA	0.12	0.62	NA	0.62	0.67	NA	1.10	1.77	0.08
		Clerks	21.60	0.63	NA	0.63	NA	0.24	0.69	NA	0.69	0.63	NA	1.08	1.71	0.09
		Service and sales workers	20.01	0.84	NA	0.84	NA	0.46	1.68	NA	1.68	0.55	NA	1.20	1.75	0.19
		Skilled agricultural and fishery workers	21.31	0.87	NA	0.87	NA	1.02	0.98	NA	0.98	0.54	NA	0.08	0.61	0.20
		Craft and related trades workers	20.64	0.89	NA	0.89	NA	0.40	0.85	NA	0.85	0.41	NA	1.66	2.07	0.15
		Plant and machine operators and assemblers	21.35	0.80	NA	0.80	NA	0.39	0.83	NA	0.83	0.54	NA	0.94	1.48	0.14
		Elementary occupations	18.80	0.86	NA	0.86	NA	0.76	2.58	NA	2.58	0.53	NA	1.16	1.69	0.20
	Finland	Legislators, senior officials and managers	23.13	0.30	0.02	0.32	0.07	0.26	0.05	0.09	0.15	0.45	0.19	NA	0.64	0.29
		Professionals	22.21	0.30	0.03	0.33	0.11	0.57	0.10	0.22	0.32	0.55	0.45	NA	1.00	0.21
		Technicians and associate professionals	21.82	0.41	0.06	0.46	0.18	0.52	0.11	0.37	0.48	0.61	0.42	NA	1.03	0.22
		Clerks	21.17	0.41	0.06	0.46	0.18	1.00	0.13	0.40	0.53	0.58	0.43	NA	1.00	0.23
		Service and sales workers	20.42	0.61	0.07	0.68	0.30	1.19	0.20	0.51	0.72	0.68	0.36	NA	1.04	0.26
		Skilled agricultural and fishery workers	20.84	0.64	0.02	0.66	0.32	0.72	0.31	0.40	0.71	0.62	0.17	NA	0.79	0.51
		Craft and related trades workers	20.35	0.57	0.05	0.61	0.18	1.34	0.26	0.31	0.56	0.70	0.24	NA	0.94	0.45
		Plant and machine operators and assemblers	20.72	0.62	0.06	0.68	0.22	1.19	0.20	0.38	0.58	0.71	0.34	NA	1.05	0.29
		Elementary occupations	19.04	0.71	0.08	0.79	0.34	2.13	0.30	0.62	0.92	0.62	0.28	NA	0.90	0.30
	Norway	Legislators, senior officials and managers	21.27	0.74	0.68	1.43	0.39	0.13	0.15	0.16	0.15	0.77	0.09	NA	0.86	0.44
		Professionals	20.76	0.77	0.79	1.56	0.50	0.12	0.30	0.42	0.72	0.66	0.22	NA	0.88	0.25
		Technicians and associate professionals	19.82	0.96	0.87	1.84	0.63	0.18	0.45	0.60	1.05	0.80	0.16	NA	0.95	0.29
		Clerks	18.99	0.82	0.70	1.52	0.78	0.32	0.91	0.80	1.72	0.82	0.15	NA	0.98	0.42
		Service and sales workers	18.17	1.35	0.84	2.19	1.12	0.25	1.16	0.98	2.14	0.41	0.08	NA	0.49	0.38
		Skilled agricultural and fishery workers	17.59	1.01	0.52	1.53	1.19	0.33	1.38	0.51	1.89	0.38	0.15	NA	0.53	1.77
		Craft and related trades workers	18.65	0.97	0.68	1.65	0.97	0.55	0.62	0.64	1.26	0.78	0.14	NA	0.92	0.66
		Plant and machine operators and assemblers	17.54	1.44	0.71	2.15	1.10	0.66	0.88	0.76	1.64	0.93	0.16	NA	1.09	0.55
		Elementary occupations	17.73	1.51	0.69	2.20	1.14	0.51	1.31	0.64	1.96	0.42	0.10	NA	0.51	0.60

Age 45	Denmark	Legislators, senior officials and managers	17.80	0.40	NA	0.40	NA	0.06	0.30	NA	0.30	0.87	NA	0.55	1.42	0.02
		Professionals	16.79	0.61	NA	0.61	NA	0.13	0.65	NA	0.65	0.61	NA	1.13	1.74	0.05
		Technicians and associate professionals	17.08	0.48	NA	0.48	NA	0.09	0.52	NA	0.52	0.67	NA	1.10	1.77	0.02
		Clerks	16.96	0.49	NA	0.49	NA	0.19	0.58	NA	0.58	0.63	NA	1.08	1.71	0.03
		Service and sales workers	15.68	0.66	NA	0.66	NA	0.33	1.43	NA	1.43	0.55	NA	1.20	1.75	0.09
		Skilled agricultural and fishery workers	16.91	0.74	NA	0.74	NA	0.84	0.81	NA	0.81	0.54	NA	0.08	0.61	0.09
		Craft and related trades workers	16.15	0.67	NA	0.67	NA	0.29	0.74	NA	0.74	0.41	NA	1.66	2.07	0.08
		Plant and machine operators and assemblers	16.79	0.62	NA	0.62	NA	0.29	0.73	NA	0.73	0.54	NA	0.94	1.48	0.09
		Elementary occupations	14.65	0.67	NA	0.67	NA	0.55	2.22	NA	2.22	0.53	NA	1.16	1.69	0.11
	Finland	Legislators, senior officials and managers	18.37	0.25	0.02	0.26	0.05	0.20	0.05	0.09	0.14	0.45	0.19	NA	0.64	0.22
		Professionals	17.52	0.25	0.03	0.28	0.09	0.42	0.09	0.22	0.31	0.55	0.45	NA	1.00	0.15
		Technicians and associate professionals	17.16	0.34	0.05	0.38	0.15	0.41	0.11	0.36	0.47	0.61	0.42	NA	1.03	0.16
		Clerks	16.73	0.34	0.05	0.39	0.15	0.74	0.12	0.39	0.51	0.58	0.43	NA	1.01	0.17
		Service and sales workers	16.04	0.51	0.06	0.57	0.25	0.91	0.19	0.51	0.70	0.68	0.36	NA	1.04	0.18
		Skilled agricultural and fishery workers	16.37	0.53	0.02	0.55	0.25	0.53	0.30	0.39	0.68	0.62	0.17	NA	0.79	0.44
		Craft and related trades workers	16.37	0.50	0.04	0.54	0.14	1.02	0.24	0.31	0.55	0.71	0.25	NA	0.96	0.32
		Plant and machine operators and assemblers	16.19	0.53	0.05	0.58	0.19	0.95	0.19	0.37	0.56	0.71	0.34	NA	1.05	0.22
		Elementary occupations	14.88	0.58	0.07	0.65	0.28	1.62	0.27	0.61	0.88	0.62	0.28	NA	0.90	0.21
	Norway	Legislators, senior officials and managers	16.85	0.58	0.54	1.12	0.32	0.10	0.15	0.15	0.15	0.78	0.09	NA	0.87	0.32
		Professionals	16.34	0.61	0.64	1.25	0.40	0.09	0.29	0.39	0.68	0.66	0.22	NA	0.88	0.18
		Technicians and associate professionals	15.59	0.75	0.69	1.44	0.51	0.14	0.42	0.57	0.99	0.80	0.16	NA	0.96	0.21
		Clerks	14.93	0.63	0.55	1.17	0.61	0.24	0.82	0.73	1.55	0.83	0.15	NA	0.98	0.31
		Service and sales workers	14.26	1.06	0.67	1.74	0.88	0.17	1.06	0.91	1.97	0.41	0.08	NA	0.49	0.27
		Skilled agricultural and fishery workers	13.86	0.81	0.41	1.22	0.89	0.18	1.27	0.47	1.74	0.38	0.15	NA	0.53	1.42
		Craft and related trades workers	14.68	0.75	0.52	1.27	0.78	0.41	0.56	0.59	1.16	0.78	0.14	NA	0.92	0.45
		Plant and machine operators and assemblers	13.66	1.12	0.56	1.67	0.87	0.52	0.81	0.74	1.55	0.93	0.16	NA	1.09	0.42
		Elementary occupations	13.92	1.20	0.56	1.77	0.94	0.34	1.20	0.60	1.81	0.42	0.10	NA	0.51	0.41
Age 50	Denmark	Legislators, senior officials and managers	12.96	0.30	NA	0.30	NA	0.04	0.26	NA	0.26	0.87	NA	0.55	1.42	0.01
		Professionals	12.14	0.44	NA	0.44	NA	0.09	0.53	NA	0.53	0.61	NA	1.13	1.74	0.02
		Technicians and associate professionals	12.38	0.34	NA	0.34	NA	0.06	0.41	NA	0.41	0.67	NA	1.10	1.77	0.01
		Clerks	12.29	0.36	NA	0.36	NA	0.13	0.46	NA	0.46	0.63	NA	1.08	1.71	0.02
		Service and sales workers	11.32	0.47	NA	0.47	NA	0.22	1.14	NA	1.14	0.55	NA	1.20	1.76	0.04
		Skilled agricultural and fishery workers	12.51	0.53	NA	0.53	NA	0.59	0.69	NA	0.69	0.54	NA	0.08	0.61	0.07
		Craft and related trades workers	11.61	0.50	NA	0.50	NA	0.22	0.57	NA	0.57	0.41	NA	1.66	2.07	0.04
		Plant and machine operators and assemblers	12.22	0.43	NA	0.43	NA	0.21	0.60	NA	0.60	0.54	NA	0.94	1.48	0.05
		Elementary occupations	10.52	0.49	NA	0.49	NA	0.37	1.79	NA	1.79	0.53	NA	1.16	1.69	0.06
	Finland	Legislators, senior officials and managers	13.57	0.19	0.01	0.20	0.04	0.15	0.05	0.08	0.13	0.45	0.19	NA	0.64	0.17
		Professionals	12.82	0.20	0.02	0.22	0.07	0.30	0.08	0.21	0.29	0.55	0.45	NA	1.00	0.11
		Technicians and associate professionals	12.52	0.26	0.04	0.30	0.11	0.30	0.09	0.34	0.44	0.61	0.42	NA	1.03	0.12
		Clerks	12.23	0.27	0.04	0.31	0.11	0.54	0.11	0.37	0.48	0.58	0.43	NA	1.02	0.13
		Service and sales workers	11.64	0.40	0.05	0.45	0.19	0.64	0.18	0.49	0.67	0.68	0.36	NA	1.04	0.13
		Skilled agricultural and fishery workers	11.90	0.42	0.01	0.43	0.16	0.39	0.28	0.36	0.64	0.62	0.18	NA	0.80	0.39
		Craft and related trades workers	11.94	0.38	0.03	0.41	0.11	0.75	0.22	0.29	0.51	0.71	0.25	NA	0.96	0.23

		Plant and machine operators and assemblers	11.69	0.41	0.04	0.45	0.15	0.70	0.17	0.36	0.53	0.71	0.34	NA	1.05	0.16
		Elementary occupations	10.80	0.45	0.06	0.51	0.21	1.15	0.24	0.60	0.84	0.63	0.29	NA	0.91	0.15
	Norway	Legislators, senior officials and managers	12.39	0.43	0.40	0.83	0.24	0.07	0.13	0.13	0.27	0.78	0.09	NA	0.87	0.23
		Professionals	12.39	0.43	0.40	0.83	0.24	0.07	0.13	0.13	0.13	0.78	0.09	NA	0.87	0.23
		Technicians and associate professionals	11.95	0.46	0.48	0.94	0.30	0.06	0.25	0.35	0.60	0.66	0.22	NA	0.88	0.12
		Clerks	11.38	0.55	0.50	1.05	0.38	0.10	0.36	0.50	0.87	0.80	0.16	NA	0.96	0.15
		Service and sales workers	10.85	0.45	0.40	0.85	0.45	0.17	0.71	0.62	1.33	0.83	0.15	NA	0.98	0.22
		Skilled agricultural and fishery workers	10.42	0.79	0.50	1.29	0.65	0.11	0.91	0.78	1.69	0.41	0.08	NA	0.49	0.19
		Craft and related trades workers	10.02	0.61	0.27	0.89	0.68	0.14	1.07	0.43	1.50	0.38	0.15	NA	0.53	1.08
		Plant and machine operators and assemblers	10.67	0.55	0.41	0.96	0.59	0.33	0.50	0.55	1.05	0.78	0.14	NA	0.93	0.32
		Elementary occupations	9.75	0.84	0.40	1.24	0.65	0.38	0.73	0.67	1.40	0.93	0.16	NA	1.09	0.29
Age 55	Denmark	Legislators, senior officials and managers	8.14	0.19	NA	0.19	NA	0.02	0.24	NA	0.24	0.87	NA	0.55	1.42	0.00
		Professionals	7.53	0.27	NA	0.27	NA	0.05	0.38	NA	0.38	0.61	NA	1.13	1.74	0.01
		Technicians and associate professionals	7.69	0.21	NA	0.21	NA	0.04	0.27	NA	0.27	0.67	NA	1.10	1.77	0.00
		Clerks	7.65	0.22	NA	0.22	NA	0.07	0.32	NA	0.32	0.63	NA	1.08	1.72	0.01
		Service and sales workers	6.97	0.29	NA	0.29	NA	0.13	0.80	NA	0.80	0.55	NA	1.20	1.76	0.01
		Skilled agricultural and fishery workers	8.25	0.41	NA	0.41	NA	0.33	0.40	NA	0.40	0.54	NA	0.08	0.61	0.01
		Craft and related trades workers	7.16	0.33	NA	0.33	NA	0.10	0.33	NA	0.33	0.41	NA	1.66	2.07	0.02
		Plant and machine operators and assemblers	7.63	0.26	NA	0.26	NA	0.13	0.45	NA	0.45	0.54	NA	0.94	1.48	0.03
		Elementary occupations	6.45	0.29	NA	0.29	NA	0.19	1.29	NA	1.29	0.53	NA	1.17	1.70	0.02
	Finland	Legislators, senior officials and managers	8.82	0.13	0.01	0.14	0.02	0.10	0.04	0.07	0.11	0.44	0.19	NA	0.63	0.11
		Professionals	8.18	0.14	0.01	0.16	0.04	0.19	0.07	0.19	0.26	0.55	0.46	NA	1.01	0.07
		Technicians and associate professionals	7.91	0.18	0.02	0.21	0.07	0.19	0.08	0.31	0.39	0.61	0.43	NA	1.04	0.08
		Clerks	7.74	0.19	0.02	0.22	0.06	0.33	0.09	0.31	0.41	0.59	0.44	NA	1.02	0.10
		Service and sales workers	7.30	0.27	0.03	0.30	0.12	0.40	0.16	0.45	0.61	0.69	0.36	NA	1.05	0.09
		Skilled agricultural and fishery workers	7.54	0.28	0.01	0.29	0.09	0.25	0.25	0.31	0.55	0.63	0.18	NA	0.80	0.31
		Craft and related trades workers	7.55	0.25	0.01	0.26	0.07	0.46	0.21	0.25	0.46	0.71	0.25	NA	0.96	0.15
		Plant and machine operators and assemblers	7.26	0.28	0.03	0.30	0.08	0.45	0.16	0.32	0.48	0.71	0.34	NA	1.05	0.11
		Elementary occupations	6.81	0.30	0.04	0.34	0.12	0.74	0.20	0.54	0.74	0.64	0.29	NA	0.93	0.10
	Norway	Legislators, senior officials and managers	7.97	0.28	0.27	0.54	0.17	0.04	0.10	0.11	0.10	0.78	0.09	NA	0.87	0.15
		Professionals	7.64	0.31	0.31	0.62	0.20	0.04	0.20	0.27	0.47	0.67	0.22	NA	0.89	0.08
		Technicians and associate professionals	7.19	0.36	0.32	0.68	0.24	0.06	0.29	0.39	0.68	0.80	0.16	NA	0.96	0.09
		Clerks	6.83	0.29	0.25	0.54	0.29	0.11	0.54	0.45	1.00	0.83	0.15	NA	0.99	0.15
		Service and sales workers	6.66	0.51	0.32	0.84	0.43	0.07	0.69	0.60	1.29	0.41	0.08	NA	0.49	0.13
		Skilled agricultural and fishery workers	6.30	0.40	0.17	0.56	0.42	0.08	0.87	0.34	1.21	0.38	0.15	NA	0.53	0.73
		Craft and related trades workers	6.71	0.33	0.27	0.60	0.37	0.21	0.43	0.47	0.90	0.79	0.14	NA	0.93	0.18
		Plant and machine operators and assemblers	6.05	0.55	0.23	0.78	0.42	0.25	0.56	0.53	1.09	0.94	0.16	NA	1.10	0.18
		Elementary occupations	6.46	0.59	0.28	0.86	0.46	0.13	0.82	0.43	1.25	0.42	0.10	NA	0.52	0.18

a. VER = Voluntary early retirement

Supplementary table S4: Reasons for working years lost (WYL) as a percentage of total WYL from age 30 to 65, among employed men and women in Denmark, Finland and Norway, by major occupational group

			Sickness absence (SA)			Time-restricted work disability	Unemployment	*Disability retirement (DR)*			*Old-age retirement (OAR)*				*Other*	*Sum*
**Age**	**Country**	**Occupational group**	*SA*	*Partial SA*	*Total*			*DR*	*Partial DR*	*Total*	*OAR*	*Partial OAR*	*VER*[a]	*Total*		
Men	Denmark	Legislators, senior officials and managers	15 %	NA	15 %	NA	5 %	6 %	NA	6 %	40 %	NA	26 %	66 %	9 %	100 %
		Professionals	14 %	NA	14 %	NA	6 %	13 %	NA	13 %	27 %	NA	25 %	52 %	13 %	100 %
		Technicians and associate professionals	13 %	NA	13 %	NA	6 %	28 %	NA	28 %	22 %	NA	22 %	44 %	8 %	100 %
		Clerks	14 %	NA	14 %	NA	10 %	24 %	NA	24 %	18 %	NA	23 %	41 %	11 %	100 %
		Service and sales workers	14 %	NA	14 %	NA	11 %	31 %	NA	31 %	10 %	NA	22 %	32 %	12 %	100 %
		Skilled agricultural and fishery workers	15 %	NA	15 %	NA	17 %	22 %	NA	22 %	17 %	NA	22 %	39 %	7 %	100 %
		Craft and related trades workers	20 %	NA	20 %	NA	12 %	18 %	NA	18 %	14 %	NA	28 %	43 %	7 %	100 %
		Plant and machine operators and assemblers	19 %	NA	19 %	NA	10 %	18 %	NA	18 %	17 %	NA	27 %	44 %	9 %	100 %
		Elementary occupations	13 %	NA	13 %	NA	17 %	35 %	NA	35 %	9 %	NA	19 %	28 %	7 %	100 %
	Finland	Legislators, senior officials and managers	14 %	1 %	15 %	3 %	13 %	4 %	4 %	7 %	28 %	10 %	NA	38 %	24 %	100 %
		Professionals	9 %	1 %	9 %	2 %	27 %	4 %	5 %	9 %	20 %	15 %	NA	36 %	17 %	100 %
		Technicians and associate professionals	12 %	2 %	13 %	3 %	24 %	6 %	5 %	11 %	23 %	11 %	NA	33 %	15 %	100 %
		Clerks	11 %	4 %	15 %	5 %	29 %	7 %	9 %	16 %	15 %	7 %	NA	23 %	13 %	100 %
		Service and sales workers	12 %	2 %	13 %	4 %	32 %	10 %	5 %	15 %	19 %	4 %	NA	23 %	13 %	100 %
		Skilled agricultural and fishery workers	15 %	4 %	20 %	5 %	20 %	11 %	8 %	19 %	16 %	4 %	NA	20 %	17 %	100 %
		Craft and related trades workers	15 %	1 %	16 %	5 %	32 %	7 %	5 %	12 %	16 %	5 %	NA	21 %	13 %	100 %
		Plant and machine operators and assemblers	17 %	2 %	19 %	5 %	26 %	9 %	5 %	14 %	19 %	6 %	NA	25 %	12 %	100 %
		Elementary occupations	12 %	3 %	14 %	4 %	41 %	10 %	5 %	15 %	13 %	4 %	NA	16 %	10 %	100 %
	Norway	Legislators, senior officials and managers	16 %	9 %	25 %	6 %	6 %	3 %	2 %	5 %	29 %	3 %	NA	32 %	26 %	100 %
		Professionals	15 %	11 %	26 %	7 %	7 %	4 %	3 %	8 %	29 %	5 %	NA	34 %	18 %	100 %
		Technicians and associate professionals	17 %	9 %	26 %	7 %	10 %	5 %	3 %	8 %	30 %	3 %	NA	33 %	16 %	100 %
		Clerks	17 %	8 %	26 %	9 %	11 %	11 %	5 %	16 %	26 %	2 %	NA	27 %	11 %	100 %
		Service and sales workers	19 %	7 %	26 %	12 %	9 %	14 %	6 %	20 %	18 %	1 %	NA	19 %	14 %	100 %
		Skilled agricultural and fishery workers	16 %	5 %	21 %	9 %	8 %	17 %	3 %	21 %	15 %	2 %	NA	16 %	25 %	100 %
		Craft and related trades workers	21 %	8 %	29 %	10 %	11 %	6 %	3 %	9 %	23 %	1 %	NA	24 %	17 %	100 %
		Plant and machine operators and assemblers	23 %	6 %	29 %	10 %	11 %	9 %	3 %	12 %	23 %	1 %	NA	24 %	14 %	100 %
		Elementary occupations	20 %	6 %	26 %	6 %	16 %	15 %	4 %	19 %	16 %	1 %	NA	17 %	16 %	100 %
Women	Denmark	Legislators, senior officials and managers	19 %	NA	19 %	NA	4 %	10 %	NA	10 %	23 %	NA	15 %	38 %	29 %	100 %
		Professionals	20 %	NA	20 %	NA	5 %	16 %	NA	16 %	12 %	NA	22 %	34 %	25 %	100 %
		Technicians and associate professionals	18 %	NA	18 %	NA	5 %	15 %	NA	15 %	14 %	NA	23 %	38 %	24 %	100 %
		Clerks	18 %	NA	18 %	NA	8 %	17 %	NA	17 %	13 %	NA	22 %	35 %	23 %	100 %
		Service and sales workers	18 %	NA	18 %	NA	12 %	29 %	NA	29 %	8 %	NA	18 %	26 %	16 %	100 %
		Skilled agricultural and fishery workers	23 %	NA	23 %	NA	25 %	22 %	NA	22 %	10 %	NA	1 %	11 %	19 %	100 %
		Craft and related trades workers	21 %	NA	21 %	NA	10 %	17 %	NA	17 %	7 %	NA	27 %	33 %	19 %	100 %
		Plant and machine operators and assemblers	23 %	NA	23 %	NA	12 %	20 %	NA	20 %	11 %	NA	19 %	30 %	16 %	100 %
		Elementary occupations	14 %	NA	14 %	NA	15 %	37 %	NA	37 %	6 %	NA	14 %	20 %	14 %	100 %
	Finland	Legislators, senior officials and managers	17 %	2 %	19 %	3 %	17 %	3 %	4 %	6 %	19 %	8 %	NA	28 %	27 %	100 %
		Professionals	11 %	1 %	12 %	4 %	28 %	3 %	7 %	9 %	16 %	13 %	NA	30 %	17 %	100 %
		Technicians and associate professionals	14 %	2 %	16 %	6 %	21 %	3 %	11 %	14 %	17 %	12 %	NA	29 %	13 %	100 %

	Clerks	12 %	2 %	13 %	5 %	34 %	3 %	9 %	12 %	13 %	10 %	NA	23 %	12 %	100 %
	Service and sales workers	14 %	2 %	16 %	7 %	33 %	4 %	10 %	13 %	13 %	7 %	NA	19 %	12 %	100 %
	Skilled agricultural and fishery workers	17 %	1 %	18 %	8 %	25 %	7 %	9 %	16 %	13 %	4 %	NA	17 %	18 %	100 %
	Craft and related trades workers	13 %	1 %	14 %	5 %	38 %	5 %	6 %	11 %	13 %	4 %	NA	17 %	15 %	100 %
	Plant and machine operators and assemblers	16 %	2 %	17 %	6 %	34 %	4 %	7 %	12 %	14 %	7 %	NA	21 %	11 %	100 %
	Elementary occupations	13 %	1 %	14 %	6 %	45 %	5 %	8 %	13 %	9 %	4 %	NA	12 %	9 %	100 %
Norway	Legislators, senior officials and managers	24 %	20 %	44 %	10 %	5 %	3 %	3 %	3 %	15 %	2 %	NA	17 %	18 %	100 %
	Professionals	22 %	21 %	43 %	11 %	4 %	6 %	8 %	14 %	13 %	4 %	NA	17 %	11 %	100 %
	Technicians and associate professionals	23 %	19 %	42 %	12 %	4 %	7 %	10 %	17 %	12 %	2 %	NA	15 %	10 %	100 %
	Clerks	17 %	13 %	30 %	14 %	7 %	13 %	11 %	24 %	11 %	2 %	NA	13 %	13 %	100 %
	Service and sales workers	23 %	13 %	36 %	17 %	5 %	14 %	12 %	26 %	5 %	1 %	NA	6 %	10 %	100 %
	Skilled agricultural and fishery workers	16 %	7 %	24 %	16 %	6 %	16 %	5 %	21 %	4 %	1 %	NA	5 %	28 %	100 %
	Craft and related trades workers	19 %	12 %	30 %	16 %	11 %	8 %	8 %	17 %	10 %	2 %	NA	11 %	15 %	100 %
	Plant and machine operators and assemblers	23 %	10 %	33 %	15 %	11 %	10 %	8 %	18 %	10 %	2 %	NA	12 %	12 %	100 %
	Elementary occupations	23 %	10 %	33 %	15 %	10 %	15 %	7 %	22 %	4 %	1 %	NA	5 %	15 %	100 %
